# Review on anti-alzheimer drug development: approaches, challenges and perspectives

**DOI:** 10.1039/d3ra08333k

**Published:** 2024-04-05

**Authors:** Abdallah E. Abdallah

**Affiliations:** a Pharmaceutical Medicinal Chemistry & Drug Design Department, Faculty of Pharmacy (Boys), Al-Azhar University 11884 Cairo Egypt abdulla_emara@azhar.edu.eg

## Abstract

Alzheimer is an irreversible progressive neurodegenerative disease that causes failure of cerebral neurons and disability of the affected person to practice normal daily life activities. There is no concrete evidence to identify the exact reason behind the disease, so several relevant hypotheses emerged, highlighting many possible therapeutic targets, such as acetylcholinesterase, cholinergic receptors, *N*-methyl d-aspartate receptors, phosphodiesterase, amyloid β protein, protein phosphatase 2A, glycogen synthase kinase-3 beta, β-secretase, γ-secretase, α-secretase, serotonergic receptors, glutaminyl cyclase, tumor necrosis factor-α, γ-aminobutyric acid receptors, and mitochondria. All of these targets have been involved in the design of new potential drugs. An extensive number of these drugs have been studied in clinical trials. However, only galantamine, donepezil, and rivastigmine (ChEIs), memantine (NMDA antagonist), and aducanumab and lecanemab (selective anti-Aβ monoclonal antibodies) have been approved for AD treatment. Many drugs failed in the clinical trials to such an extent that questions have been posed about the significance of some of the aforementioned targets. On the contrary, the data of other drugs were promising and shed light on the significance of their targets for the development of new potent anti-alzheimer drugs.

## Abstract 2

Alzheimer has many crucial factors that should be considered in order to get better results from clinical trials. Benzimidazole and its isosteres represent significant scaffolds for designing potential multi-target anti-alzheimer molecules.

## Graphical abstract

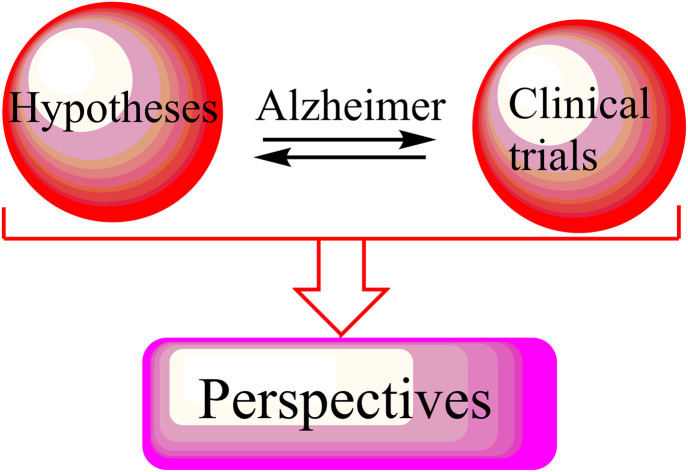

## 1. Introduction

### 1.1. Background

Alzheimer disease (AD) is an irreversible and progressive neurodegenerative disorder leading to ultimate damage and death of brain neurons.^1,2^ It causes cognitive impairment, behavioral defects, psychological disorders, memory loss, and disturbances in daily life activities such as eating, drinking, reading, writing, walking, communication, *etc.*^3^ It significantly affects not only the life of patients, but also that of their family.^4^ It mainly affects old people at or above 65 years old, but nowadays it seems to touch younger adults as a consequence of modern lifestyles.^3^ AD is the primary cause of dementia and affects approximately 10% of people over the age of 65 and 50% over the age of 85.^5^ It was reported that 6.7 million American people lived with clinical AD in 2023, and this figure is expected to reach 8.5 million by the year 2030.^6^ In a separate report, the estimate of worldwide people with AD reaches 74.7 million, with a care cost of about US$2 trillion by 2030.^5^

### 1.2. Etiology

Unfortunately, the etiology of AD is still incompletely understood. There is no definite reason evident to be behind the disease. Alternatively, AD is considered to be related to different reasons, among which are genetic factors and environmental effects such as mental stress, food habits, and lifestyle.^7^ However, histopathological studies revealed relevant multifactorial disorders from which different hypotheses originated to shed light on the likely mechanisms and effective targets of the disease. The emerged hypotheses include the following: (a) cholinergic hypothesis, (b) amyloid cascade hypothesis, (c) tau hypothesis, (d) mitochondrial cascade hypothesis, (e) oxidative stress hypothesis (f) excitotoxicity hypothesis, (g) neuroinflammatory hypothesis, and (h) others (like genetic factors, environmental factors, *etc.*).

#### 1.2.1. Cholinergic hypothesis

It is evident that the whole central neurotransmitter system is affected in AD; however, the cholinergic system remains comparatively the most deteriorated one.^8–10^ Furthermore, a correlation between the central cholinergic deficit and the degree of cognitive disorder was detected.^11–13^ Because ACh was found to be involved in cognitive processes, work on increasing ACh levels gained a lot of attention in order to restore cognitive normality (the so-called cholinergic hypothesis). Consequently, many drugs have been developed as acetylcholinesterase inhibitors (AChEIs) in order to increase the ACh levels in the brain.^14^ It is obvious that such drugs do not repair the damaged neurons; instead, they alleviate the symptoms by increasing the levels and duration of action of the central ACh. Accordingly, they can be described as symptomatic therapy without curing or even preventing the progression of the disease.^13,15^ In such a case, new perspectives on disease control appear to be highly reasonable. As a consequence, some other mechanisms and targets were proposed, as we can see below.

#### 1.2.2. Amyloid cascade hypothesis

One of the most characteristic pathological hallmarks in AD brains is extracellular senile plaques, which lead to neuronal damage, pathogenesis, and disease progression.^16^ It was discovered that the amyloid β protein (Aβ) is the primary component of the amyloid plaques in AD.^17^ In general, there are three forms of Aβ: soluble monomer, soluble oligomer, and insoluble fibril, which are normally degraded and released away from the neurons.^18^ Furthermore, amyloid precursor protein (APP) plays a crucial role in neurite development and neuronal membrane trafficking.^18^ However, in AD, two enzymes (β- and γ-secretase) were identified to be involved in the overproduction of insoluble Aβ by cleaving APP. This process comprises two successive steps. β-Site amyloid precursor protein cleaving enzyme-1 (BACE1), the main form of β-secretase in the CNS, mediates the first step by cleaving APP to sAPPb and membrane bound C-terminal APP fragment (C99).^19^ The latter is then cleaved by γ-secretase to give Aβ peptides, which include Aβ40 and Aβ42. Due to a genetic mutation, Aβ42 is overproduced in AD. It was found that Aβ42 is hydrophobic and tends to accumulate, forming amyloid plaques rather than Aβ40.^20,21^ Relatively high levels of BACE1 were detected in sporadic AD brains, accelerating the first step (the rate-limiting one) in the generation of Aβ from APP.^22,23^

These facts draw attention to amyloid cascade hypothesis as a considerable theory of AD pathogenesis.^24^ Over decades, researchers considered Aβ aggregation as the main cause of all AD pathological changes such as neurotoxicity, neuronal inflammation, and neuron loss.^16,18^ Accordingly, several attempts at developing anti-Alzheimer drugs targeting Aβ have been made.^25,26^ But repeated failures of Aβ-targeted clinical trials pose a question concerning the significance of the amyloid hypothesis.^16^ On the basis of such deficiencies in the amyloid hypothesis as well as some recent findings, researchers suggested that the major factor underlying the development and progression of AD is tau rather than Aβ.^27^

#### 1.2.3. Tau hypothesis

Tau is one of the microtubule-associated proteins that regulate the stability of tubulin assemblies.^27^ Pathological hyperphosphorylation of tau is observed in AD brains, causing the accumulation of phosphorylated tau inside neuronal cells in the form of neurofibrillary tangles that eventually cause neuronal death.^27–29^ Hyperphosphorylated tau is cytotoxic. It inhibits the assembly and functions of tubulin, disrupting proper intracellular transportation. In addition, it negatively affects the integrity of the mitochondrial membrane, leading to mitochondrial swelling and functional defects.^18,29,30^ Tau hyperphosphorylation is correlated to dysfunction of glycogen synthase kinase-3 beta (GSK-3β) and/or protein phosphatase 2A (PP2A).^31,32^ GSK-3β and PP2A are enzymes involved in the regulation of tau phosphorylation.^32,33^

In contrast to amyloid plaques that aggregate extracellularly, tau-based neurofibrillary tangles are accumulated intracellularly. It was proposed that tau pathology is the crucial factor and occurs earlier than Aβ aggregation in sporadic AD.^29^ So, the tau hypothesis is likely to reveal more predominant effects on neurons than the amyloid hypothesis.^27^

##### 1.2.3.1. Mitochondrial hypothesis

Mitochondrial function changes over time under the effects of genetic and/or environmental factors. Oxidative stress is a fundamental element that disrupts mitochondrial function and induces mitochondrial fragmentation during the pathogenesis of AD.^34^ The mitochondrial hypothesis states that in sporadic, late-onset AD, changes in mitochondrial function affect APP expression and amyloid accumulation in a manner that triggers the pathogenesis of amyloid cascade.^35^ Correlations have been increasingly recognized between mitochondrial function, Aβ amyloidosis, and tau phosphorylation.^36^

#### 1.2.4. Oxidative stress hypothesis

However, sporadic AD is not linked to genetic mutation; it was suggested to be likely associated with the pathological role of oxidative stress raised by abnormal metabolic reactions in the CNS.^37^ The pathological hallmarks of the disease, such as amyloid plaques and neurofibrillary tangles, are a result of abnormalities in protein metabolism. This contributes to the oxidative stress in the sense that Aβ was found to generate free radicals.^37^ Furthermore, there is some evidence that supports increased oxidative stress in AD brain, such as increased levels of Fe, Al, and Hg that can generate free radicals. In addition to increased lipid peroxidation and decreased polyunsaturated fatty acids, there is also increased protein and DNA oxidation in the AD brain. Moreover, diminished energy metabolism and decreased cytochrome c oxidase were observed in AD brain.^37^ Accordingly, AD brains exhibit constant evidence of reactive oxygen species (ROS) and reactive nitrogen species (RNS)-mediated injury.^38^

#### 1.2.5. Excitotoxicity hypothesis

Overstimulation of *N*-methyl d-aspartate (NMDA) receptors by endogenous glutamate (Glu) causes excitotoxic neuronal degeneration in acute central nervous system injury syndromes such as stroke and trauma.^39,40^ Similarly, continuous mild activation of NMDA leads, under chronic conditions, to neuronal damage.^41^ Furthermore, neural plasticity is likely to be impaired not only from neuronal damage but also from continuous activation of NMDA receptors.^41^ In AD, this disorder originates primarily from the toxic effects of accumulated Aβ on certain synapses, targeting the glutamate receptors NMDA and 2-amino-3-(3-hydroxy-5-methyl-isoxazol-4-yl)propanoic acid (AMPA). The consequences of this effect are the dysregulation and reduction of expression of NMDA and AMPA, and hence the accumulation of the excitatory amino acids glutamate and d-serine. Eventually, this results in synapse failure in AD.^42^ To date, synapse failure is considered one of the primary pathological markers linked to cognitive decline and AD pathogenesis.^43,44^ Some researchers refer to this mechanism as synaptic failure hypothesis.^45^

#### 1.2.6. Neuroinflammation hypothesis

It is evident that AD is linked to neuroinflammation, which is triggered by several factors during the disease progression. High levels of proinflammatory cytokines such as tumor necrosis factor alpha (TNF-α) and interleukin-6 (IL-6) are detected in AD brain.^46^ Furthermore, activated microglial cells and astrocytes were observed in AD brain.^47^ It was suggested that activated microglial cells stimulate the release of proinflammatory mediators, leading to neurotoxicity, neuronal damage, and impairment of Aβ clearance.^47,48^ Activation of microglial cells occurs as a response to some chronic disorders in the brain, among which are amyloid plaques.^48,49^ So, accumulation of microglial cells was detected at the precipitated Aβ.^50^ Additionally, neuronal overexpression of cyclooxygenase-2 was identified at different stages of AD.^51^ Some studies have revealed that neuroinflammation is a relatively early pathological feature of AD.^51^

#### 1.2.7. Genetic factor of AD

In familial AD, genetic mutations in APP, presenilin 1, and presenilin 2 have been recognized. γ-Secretase, an enzyme linked to overproduction of insoluble Aβ, is encoded by presenilin 1 and presenilin 2.^52^ In sporadic AD, polymorphism in multiple genes has been identified.^53^ Among them is polymorphism in the ε4 and ε2 variants of the apolipoprotein E (APOE) gene.^54^ So, APOE is considered one of the most fundamental risk factors of sporadic AD.^55^ Some studies revealed that the genetic factor accounts for about 80% of AD.^54^

### 1.3. Challenges

There are some challenges in the treatment of AD, such as a lack of evidence of the exact mechanism and the primary target on which researchers can work.^56^ Furthermore, no neuronal protective or regenerative drug is available nowadays.^57^ There is no cure for damaged neurons, but there are attempts to stop or delay the worsening of AD. The development of AD drugs was based on different hypotheses that shed light on the histopathological disorders in AD. However, the results of clinical trials questioned almost all of these hypotheses and revealed that none of them can be considered the sole approach to treatment. The data from clinical trials also reflect the complexity of the disease, which has different essential factors contributing to its pathogenesis. Furthermore, AD takes a relatively longer time to complete the clinical study in comparison to most other therapeutic fields.^58^ One more challenge is that AD is a progressive disease. Each stage may require its own specific study because early-stage drugs are likely to be ineffective in later stages. AD is also chronic, which reflects the requirement of drug safety for long-term use.

However, there are advances in anti-alzheimer drugs and their mechanisms of action. The following section concerns the drugs approved for the treatment of AD, and the drugs passed to clinical trials. The results of these clinical studies and their significance are shown below, considering the mechanisms of action extended to a wide range of targets identified in this review.

## 2. Discussion

In terms of their mechanisms of action, anti-alzheimer drugs can be classified as the following.

### 2.1. Cholinergic drugs

#### 2.1.1. ChEI

On the basis of the cholinergic hypothesis, choline esterase inhibitors have been developed in order to improve the symptoms of AD. Two types of enzymes have been employed: acetylcholinesterase (AChE) and butyrylcholinesterase (BChE). BChE was found to distribute in the brain and have the ability to degrade ACh. There is some evidence that BChE plays a major role in the hydrolysis of ACh and compensates for the function of AChE when its level is decreased or its production is inhibited in advanced AD.^59,60^ In fact, in advanced AD, AChE level declines to 90% in comparison to the levels in a healthy brain. On the other hand, the level of BChE was found to continuously increase in advanced AD.^61^

Three drugs are currently FDA-approved for the treatment of AD as ChEIs: galantamine, donepezil, and rivastigmine. Donepezil and galantamine are selective AChEIs,^62^ while rivastigmine has a dual inhibitory effect on AChE and BChE.^63^

##### 2.1.1.1. AChEIs

**Fig. 1:**
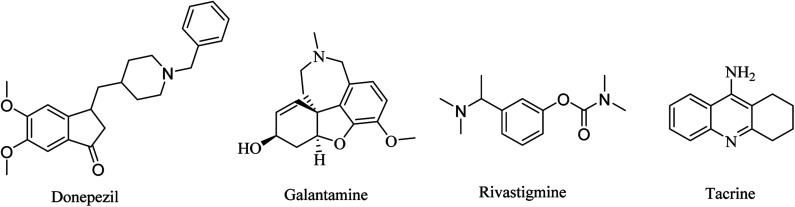
The chemical structures of approved cholinesterase inhibitors.

Galantamine is a natural alkaloid based on benzofuro[4,3-*cd*]azepine (Fig. 1). It is a reversible AChE inhibitor with no effect on BChE. Furthermore, its cholinergic activity is enhanced by binding to allosteric nicotinic sites. It is exposed to minor biotransformation that includes demethylation of about 5–6% of the dose. It is excreted mainly in the urine.^64^

Donepezil is another reversible AChE inhibitor that is based on an inden-1-one nucleus (Fig. 1). It is indicated for symptomatic treatment of patients suffering from mild-to-moderate AD. Donepezil is extensively bound to plasma proteins (about 96%), so its elimination half-life is prolonged to 70 h.^64^

##### 2.1.1.2. Dual AChE and BChE inhibitors

Rivastigmine is a pseudoirreversible noncompetitive carbamate inhibitor of AChE and BChE (Fig. 1). Although its half-life is limited to only 2 h, its inhibition of ChEs is extended to approximately 10 h. This prolonged effect is attributed to the slow dissociation of the drug enzyme complex. In April 2000, rivastigmine was approved by the Food and Drug Administration (FDA) for the treatment of mild-to-moderate AD.^64^

Tacrine is another dual AChE and BChE inhibitor, based on an acridine nucleus (Fig. 1). Due to its hepatotoxicity, it has been withdrawn from the market.^65^ This class of drugs represents just a symptomatic treatment without preventing the progression of the disease.^66–69^

**Fig. 2:**
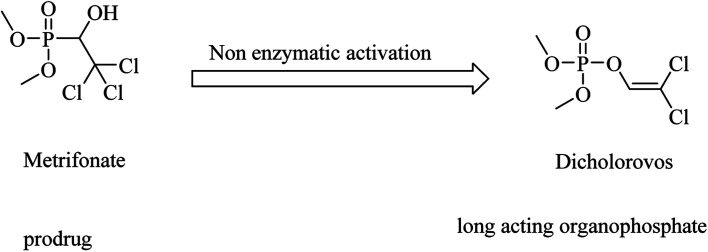
Chemical structures of organophosphate molecules as cholinesterase inhibitors.

Metrifonate, dimethyl (2,2,2-trichloro-1-hydroxyethyl)phosphonate, is an irreversible inhibitor for both ChEs, with higher selectivity for BChE than AChE. It is a prodrug converted nonenzymatically to the active metabolite dichlorvos, which is responsible for the sustained cholinesterase inhibition (see Fig. 2). Its clinical evaluation in mild-to-moderate AD revealed its toxicity, so its use was suspended for AD patients.^64^

#### 2.1.2. Nicotinic receptor agonist

A different approach to developing new selective anti-alzheimer drugs was targeting the α7 nicotinic receptor instead of acting on all cholinergic receptors, in the sense that this receptor plays a significant role in memory, learning, and executive functions. In contrast to currently marketed AChE inhibitors, which are known to have considerable side effects, the new approach is more likely to eventually develop drugs of relatively high safety.^70,71^

**Fig. 3:**
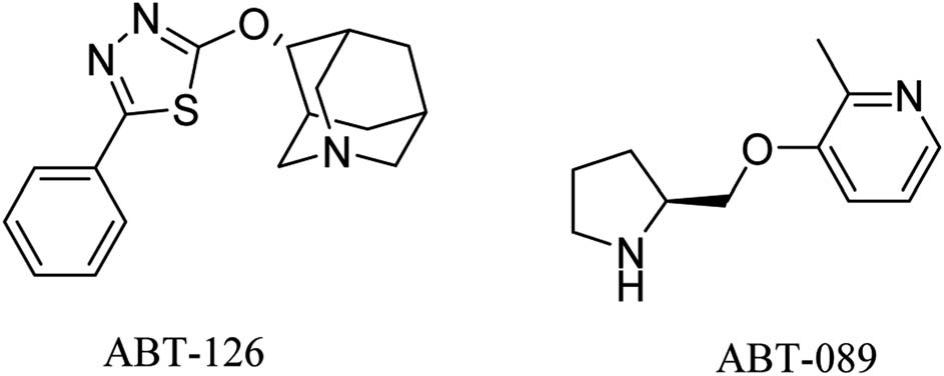
The chemical structures of some nicotinic receptor agonists.

ABT-126 (Fig. 3) was identified as a selective α7 nicotinic receptor agonist and suggested as a monotherapy in mild to moderate AD. In phase 2a clinical trials, ABT-126 improved cognition to some extent in patients with mild to moderate AD, while it failed in phase 2b study to reveal any significant improvement^72^ and showed no therapeutic effect.^73^ With respect to safety profile, ABT-126 was generally well tolerated.^73^

Similarly, α4β2 neuronal nicotinic receptors are associated with cognitive functions such as learning, memory, and attention.^74^ ABT-089 (Fig. 3) was developed as a selective α4β2 partial agonist,^75^ but it showed no therapeutic effects against alzheimer in clinical trials.^76^

#### 2.1.3. Muscarinic receptor agonists

**Fig. 4:**
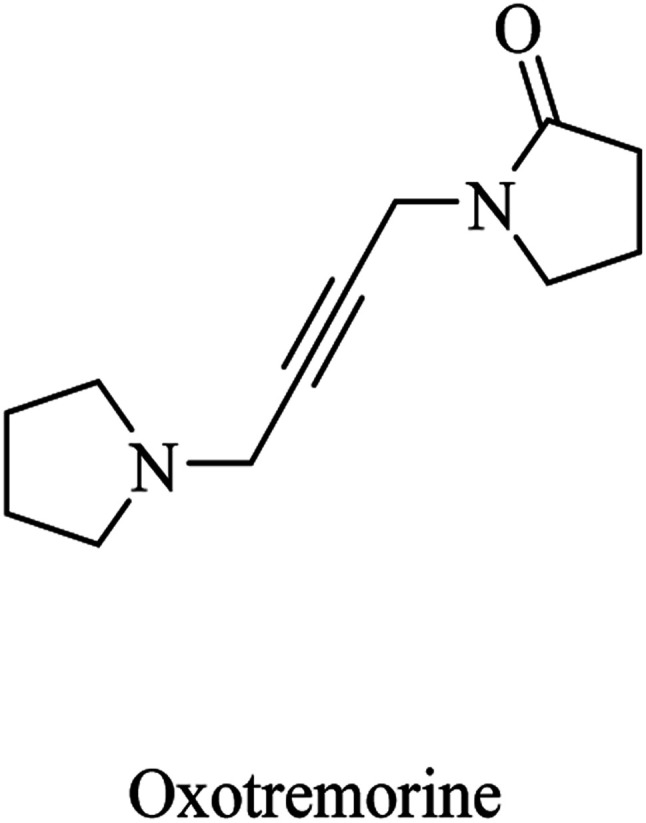
Oxotremorine chemical structure.

Oxotremorine (Fig. 4) was developed as a stimulant for CNS muscarinic receptors. The evaluation of the drug as a potential treatment for AD revealed that oxotremorine increased the levels of ACh up to 40% in the rats' brains. Despite this promising effect that appears to help AD patients, the data from other studies were disappointing. So, the usefulness of oxotremorine in AD is highly disputed by many researchers.^77^

**Fig. 5:**
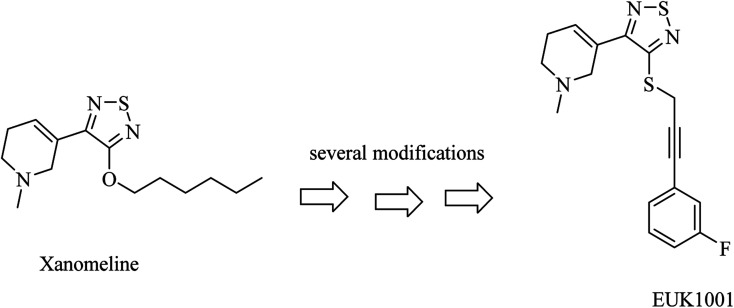
Development of EUK1001 as a xanomeline analog.

Xanomeline is a selective muscarinic agonist based on the pyridinylthiadiazole scaffold (Fig. 5). It showed a significant improvement in AD patients in clinical trials.^78^ On the other side, it triggered a lot of unwanted side effects. However, it was considered a lead for the development of anti-alzheimer drugs.^79^

EUK1001 is a fluorinated derivative of xanomeline, as can be noticed from Fig. 5. EUK1001 was found to decrease AD-like neurodegenerative disorder in presenilin-deficient mice, which has no Aβ pathology. Furthermore, EUK1001 revealed a significant improvement in cognitive functions in AD mice as well as a reduction in Aβ42.^79^ Meanwhile, another study indicated that EUK1001 improved memory function in aged mice.^80^ According to these data, EUK1001 was suggested as a promising compound for the treatment of AD.^81^

### 2.2. Glutamatergic drugs

**Fig. 6:**
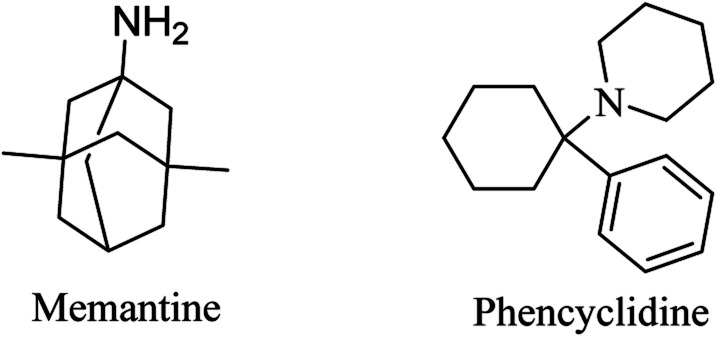
The chemical structure of some glutamatergic drugs.

Memantine (Fig. 6), an adamantine derivative, was approved by the FDA, and hence it is currently used for the treatment of AD as an NMDA antagonist.^82^ It clinically enhances cognitive ability and improves behavioral disturbance with an excellent safety profile, whether it is used alone or in combination with donepezil.^83–85^ Memantine blocks the NMDA receptor, reducing calcium ion influx into the neurons, so that it prevents the activation of toxic intracellular events.^40^ It was found to have a low-to-moderate affinity for NMDA.^86^ On the other side, antagonists with high affinity, such as phencyclidine (Fig. 6), revealed severe adverse effects that make their use for alzheimer not practical.^86^

### 2.3. Phosphodiesterase inhibitors (PDEIs)

Inhibition of PDE was suggested to prevent and improve AD by increasing the levels of cyclic guanosine monophosphate (cGMP) and cyclic adenosine monophosphate (cAMP).^87^

#### 2.3.1. PDE1 inhibitors

**Fig. 7:**
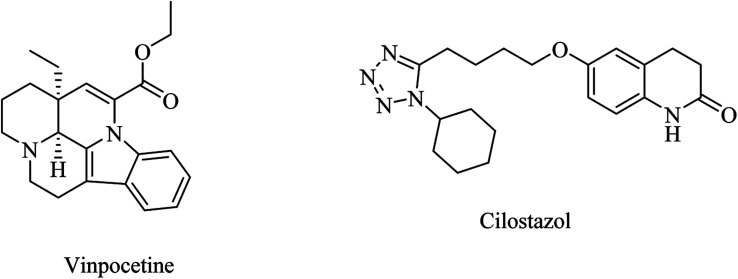
The chemical structures of vinpocetine and cilostazol.

Vinpocetine (Fig. 7) is a PDE1 inhibitor.^88^ In Europe, it has been approved for the treatment of dementia.^89^ Its preclinical data indicated some significant effects, such as repairing cognitive impairment in a rodent AD model,^90^ downregulating BACE1,^90^ decreasing oxidative stress,^91,92^ and reducing mitochondrial dysfunction.^93^ Despite the significant preclinical findings, the results of clinical studies were disappointing with regard to the improvement of AD patients.^94^

#### 2.3.2. PDE3 inhibitors

Cilostazol, a 6-substituted quinolinone (Fig. 7) PDE3 inhibitor, showed inconclusive clinical trials in terms of efficacy in the treatment of AD.^87^

#### 2.3.3. PDE4 inhibitors

**Fig. 8:**
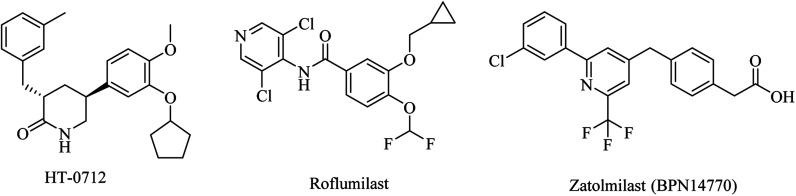
The chemical structures of some PDE4 inhibitors.

On the other hand, PDE4 inhibitors were promising to a great extent.^95^ HT-0712, a 3,5-disubstituted piperidinone (Fig. 8), has completed a phase II study, showing enhancement in the long-term memory of patients with age-related memory impairment.^96^ It is now under another clinical trial study designed to further evaluate its effect on AD improvement.^97^

Meanwhile, a phase I study showed that a combination of roflumilast, a *N*-pyridinylbenzamide-based molecule (Fig. 8), and donepezil improved verbal learning performance.^96^ In a recent study, roflumilast reduced neuroinflammation, amyloidogenesis, oxidative stress, cholinergic impairments, and phosphorylated tau levels in the rat hippocampus exposed to streptozotocin-induced sporadic AD.^98^

Zatolmilast (BPN14770), a phenylacetic acid derivative (Fig. 8), was reported as a PDE4D-negative allosteric inhibitor. It was found to show an improvement in memory and cognitive functions. Two clinical studies revealed that BPN14770 is safe and well tolerated. It was designed not to completely inhibit the enzyme in order to reduce the emetic effect.^96^

**Fig. 9:**
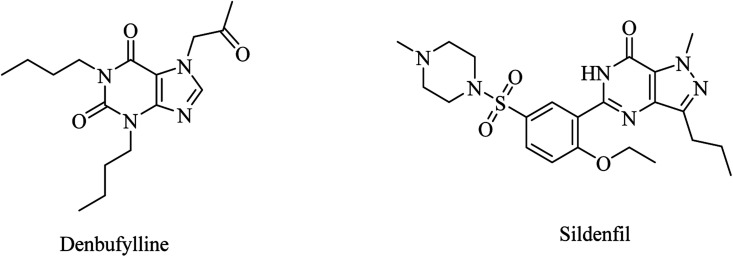
The chemical structure of denbufylline and sildenafil.

Clinical trials of denbufylline, a xanthine PDE4 inhibitor (Fig. 9), were inconclusive and preliminary.^99^

#### 2.3.4. PDE5 inhibitors

Sildenafil, a pyrazolopyrimidine-based PDE5 inhibitor (Fig. 9), showed improvement in cognitive functions, but these promising results are still preliminary and inconclusive.^87,96^

#### 2.3.5. PDE9 inhibitors

**Fig. 10:**
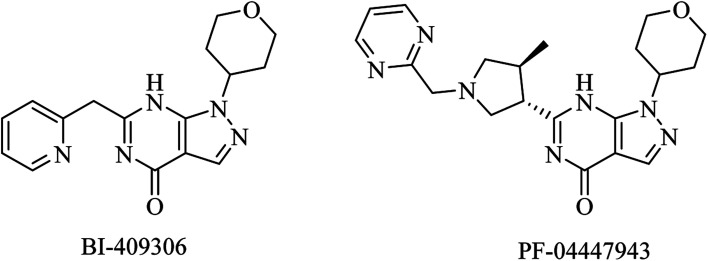
The chemical structure of some PDE9 inhibitors.

BI-409306 and PF-04447943 are other pyrazolopyrimidine derivatives (Fig. 10) that were developed as potent and selective PDE9 inhibitors.^100,101^ However, their clinical trials revealed no significant effect on AD.^100,101^

#### 2.3.6. Broad spectrum PDE inhibitors

**Fig. 11:**
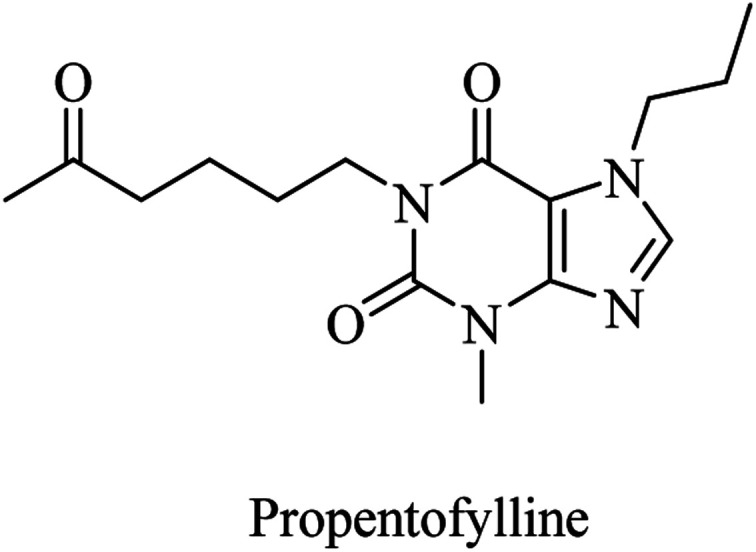
Xanthine based broad spectrum PDEI.

Propentofylline, a xanthine derivative (Fig. 11), is a broad-spectrum PDE inhibitor. Five phase III studies on it revealed enhancement in cognitive functions, reduction in dementia severity, and improvement in daily life activities in mild-to-moderate AD patients. However, two books claimed, based on a MedScape article, that an 18 months phase III trial failed, so it was discontinued.^87^

### 2.4. Anti-Aβ drugs

Anti-Aβ drugs have been designed to degrade the amyloid plaques either chemically or immunologically by activating phagocytosis or microglia. This is likely to prevent the neuronal damage triggered by the amyloid plaques.^57,102–104^

#### 2.4.1. Immunotherapy

The most studied approaches were active and passive immunotherapies. In fact, nearly a dozen anti-Aβ monoclonal antibodies have been developed and clinically studied for AD.^105^

##### 2.4.1.1. Passive immunotherapy

For years, a lot of molecules have been designed and evaluated for their effects on senile amyloid plaques as a significant hallmark of AD pathogenesis. Many therapeutic targets directly or indirectly linked to Aβ protein have been involved. As a result of such a huge effort, aducanumab and lecanemab were approved by the FDA.^57,106,107^

Aducanumab is a selective anti-Aβ monoclonal antibody with the ability to clear Aβ plaques. On the basis of these proven data, the FDA approved it in June 2021 as the first drug underlying the pathophysiology of Alzheimer's disease (AD). On the other side, the correlation between clearance of Aβ plaques and improvement in cognitive functions lacks evidence. Moreover, the data obtained from two phase III studies were controversial and insufficient to prove aducanumab efficacy. So, the accelerated approval of aducanumab by the FDA generated a debate among scientists. Some researchers consider this approval an obstacle to progress and pose a question concerning the cost and safety profile of aducanumab. A further argument in support of the insignificance of aducanumab is the rejection of it by the European Medicines Agency in December 2021. Now, Biogen is designing a confirmatory study, named ENVISION, required by the FDA, and it is expected to be complete in 2026.^108–112^

Lecanemab is a monoclonal antibody directed against both soluble and insoluble forms of Aβ polypeptides.^113^ Lecanemab received FDA-accelerated approval in January 2023.^113–116^ After further investigation, it received traditional approval from FDA in July 2023 for the treatment of early-stage AD.^106^ Lecanemab showed both amyloid clearance and a slowing of clinical decline in early AD.^117^

On the contrary, solanezumab,^118,119^ bapineuzumab,^120,121^ ponezumab,^122^ and gantenerumab^119,123,124^ failed in clinical trials as monoclonal anti-Aβ antibodies.^57^ Similarly, clinical trials on the natural anti-Aβ antibodies obtained from the blood of healthy adults or Alzheimer patients revealed no clinical effects on AD.^57^

##### 2.4.1.2. Active immunotherapy

One important aspect of immunotherapy is vaccination. It takes successive stages of clinical trials. Firstly, clinical trials of vaccines composed of purified Aβ-42 polypeptide (AN1792)^125^ were disappointing due to the toxicity detected in about 6% of AN1792-treated patients, such as cytotoxic T-cell-induced meningoencephalitis.^126,127^ Furthermore, AN1792 failed to clear amyloid plaques; however, it activated the production of Aβ antibodies in AD patients' blood.^128^ Due to the detected toxicity linked to the full-length Aβ1-42 vaccine, the concept has been modified to develop a new generation such as vanutide cridificar (ACC-001) that was designed to stimulate antibodies against N-terminal Aβ1-7. The results obtained were acceptable with regard to safety but showed no therapeutic effects.^57^ Therefore, the next clinical trials involved a combination of ACC-001 and QS-21 adjuvant. The results revealed a good safety profile and high levels of anti-amyloid beta IgG in mild to moderate AD patients after long-term exposure to the combination.^129,130^ This high level of anti-amyloid-beta IgG declined after some time.^131^ In another trial, ABvac40 vaccine was designed to initiate the production of antibodies against the C-terminal end of Aβ40. It showed good tolerability and developed Aβ40 specific antibodies.^57^ ABvac40 phase II clinical studies are still being processed.^132^

#### 2.4.2. Small molecules

**Fig. 12:**
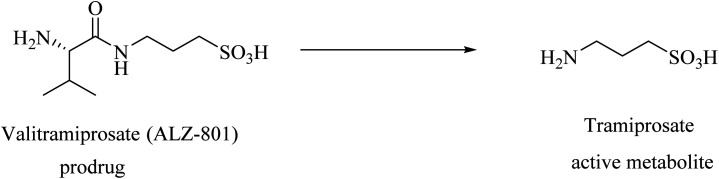
Tramiprosate and its optimized prodrug valiltramiprosate.

Tramiprosate, 3-aminopropane-1-sulfonic acid (Fig. 12), is an anti-amyloid oral small molecule that revealed promising clinical results. It was found to selectively and strongly inhibit Aβ42 oligomer formation and the subsequent amyloid aggregation without binding to plaques.^133–136^ In a phase 2 study in AD patients, tramiprosate was found to pass the BBB and reduce Aβ42 levels in a dose-dependent manner.^137^ In a phase III clinical study conducted on mild to moderate AD patients, tramiprosate showed significant efficacy in APOE4/4 homozygotes, and intermediate efficacy in APOE4 heterozygotes. But no effects have been observed on non-carrier patients.^137^ The observed side effects were nausea, vomiting, and weight loss.^137^ Indeed, further large and controlled trials are required to confirm the significant results of tramiprosate.^135^

Valiltramiprosate (ALZ-801) was designed as an optimized prodrug (Fig. 12) to address the extensive gastrointestinal metabolism and gastrointestinal irritation detected for the active drug tramiprosate.^133^

Phase I clinical trials of ALZ-801 revealed good tolerability and no serious side effects.^133^ ALZ-801 was also found to pass across the blood–brain barrier (BBB) efficiently, showing an intracranial concentration of about 40% of its plasma levels.^138^ ALZ-801 phase II and phase III studies on APOE4 carries are ongoing.^139^

### 2.5. Tau protein targeting drugs

Tau protein represents a crucial factor in AD pathogenesis. Nevertheless, the benefits of clinical trials on molecules targeting tau hyperphosphorylation were insignificant in terms of efficacy and/or safety.^57^

#### 2.5.1. PP2A activators

One of the fundamental regulators of tau phosphorylation is PP2A. It acts directly or indirectly by affecting GSK-3β.^33^ So, it was a promising target for the development of potential anti-alzheimer drugs.

In addition to its action as an NMDA antagonist, memantine (Fig. 6) was found to activate PP2A, leading to inhibition of tau hyperphosphorylation.^140^

Sodium selenate is another phosphate modifier that increases the activity of PP2A.^140^ The data of a phase IIa clinical trial, in patients with mild to moderate AD, revealed some benefits for sodium selenate on diffusion MRI. But it failed to show any considerable improvement in other measures such as cognition and CSF levels of Aβ and tau proteins.^141^

**Fig. 13:**
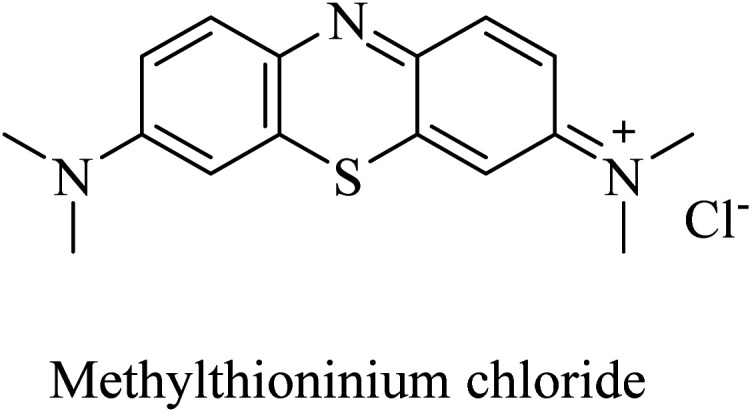
The chemical structure of MTC.

Methylthioninium chloride (MTC) (Fig. 13), known as methylene blue, was the first molecule considered to inhibit tau aggregation without affecting the physiological function of tau as a stabilizer for neuronal microtubules.^142^ On the other side, the poor pharmacokinetics and intolerability of MTC were likely to be the reasons behind inefficacy in phase 2 clinical trials.^57^

#### 2.5.2. GSK-3β inhibitors

The regulation of tau phosphorylation is primarily achieved by a balance between the two opposite proteins tau kinase and phosphatase activities. Tau hyperphosphorylation is suggested to be triggered when this balance is disrupted.^143^ Accordingly, tau kinases emerged as interesting targets for the development of anti-alzheimer drugs.^143^ In particular, the overexpression of GSK3β was found to initiate tau phosphorylation and trigger neurodegeneration.^144^

**Fig. 14:**
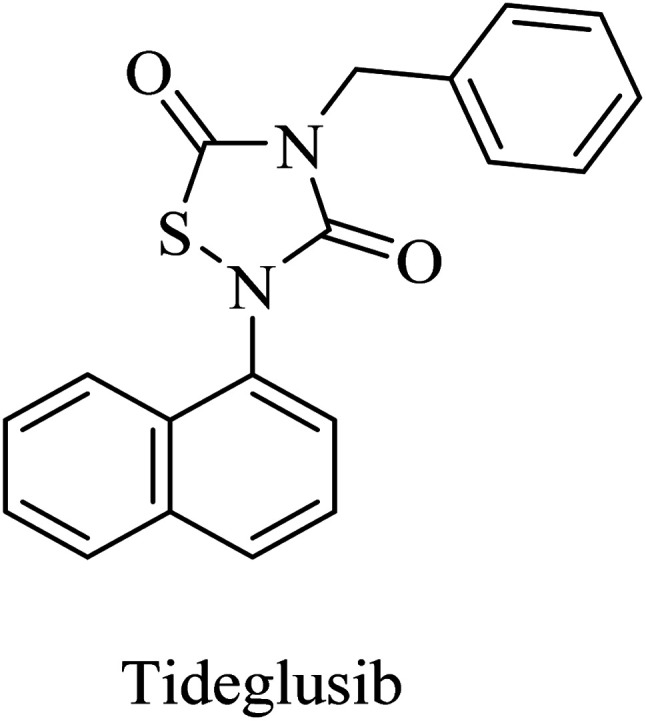
Structure of tideglusib.

Tideglusib, a thiadiazolinedione derivative (Fig. 14), is developed to irreversibly inhibit GSK3β without interaction with the ATP binding domain.^145,146^ In animal models of AD, it caused a reduction in neurofibrillary tangles and amyloid plaques with an improvement in memory.^147,148^ These data were confirmed by the preliminary data of a larger-scale phase II clinical trial in which tideglusib caused an improvement in cognitive functions along with a reduction in CSF β-secretase levels in a subgroup of mild AD patients. However, the precise analysis of the entire study revealed the insignificance of the improvement caused by tideglusib.^149^

Lithium chloride (or ‘lithium’) was identified as a GSK3β inhibitor.^150^ Preliminary results of lithium clinical trials showed some improvement with few side effects in elderly patients with AD. Further studies are required to evaluate the efficacy and safety of lithium in AD patients.^140^

### 2.6. β-secretase acting drugs

#### 2.6.1. BACE-1 inhibitors

**Fig. 15:**
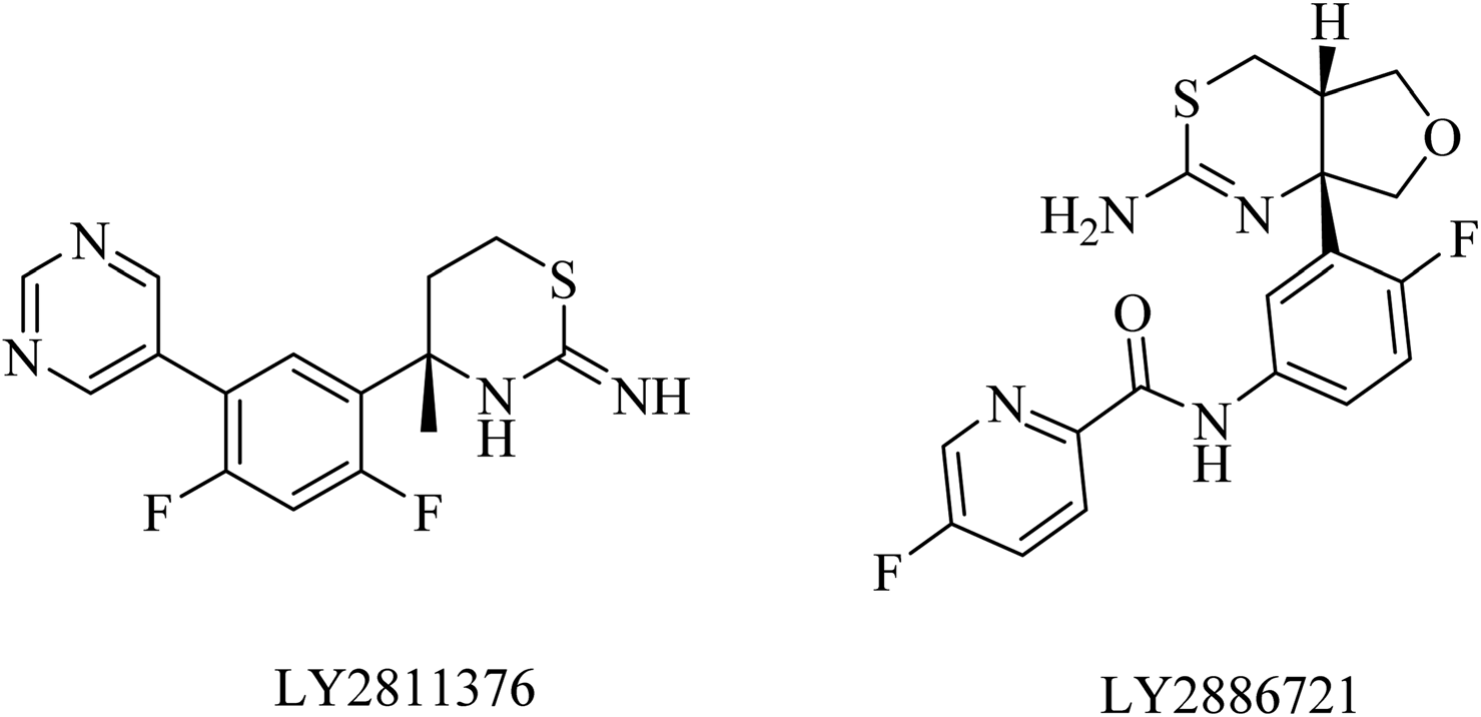
The chemical structures of some BACE1 inhibitors.

LY2811376 [(*S*)-4-(2,4-difluoro-5-pyrimidin-5-yl-phenyl)-4-methyl-5,6-dihydro-4*H*-[1,3]thiazin-2-yl-amine] (Fig. 15) was constructed through fragment-based drug design as an oral BACE1 inhibitor. It showed good oral bioavailability and promising effects in animal models, which reflects its safety and tolerability in healthy volunteers. Meanwhile, subsequent oral doses of 30 or 90 mg of LY2811376 revealed a considerable and long-term reduction in Aβ levels in CSF. In a dose dependent manner, an increase in Aβ5-40 and a decrease in Aβ1-34 were associated with the use of LY2811376 in AD.^151^ In contrast to these promising pharmacodynamic features, its long-term use in preclinical studies showed toxic effects, which prevented any further progress in the clinical trials, and accordingly, the drug was discontinued in phase II.^152^

LY2886721 is a picolinamide-based (Fig. 15) BACE1 inhibitor. Its oral administration in a transgenic mouse model-based study showed a significant reduction in the levels of Aβ and soluble APPβ (sAPPβ). Similar promising results were obtained in another study that revealed a great and continuous reduction in the levels of Aβ in CSF after oral use of LY2886721 in a cannulated beagle dog model. In addition to this, LY2886721 was found to have good BBB penetration, showing high CSF concentrations.^16,153^ The drug LY2886721 has been exposed to clinical trials because of its good pharmacology and safety profile. In a phase II clinical study, LY2886721 induced an abnormal elevation in liver enzymes, so the study was terminated.^154^

**Fig. 16:**
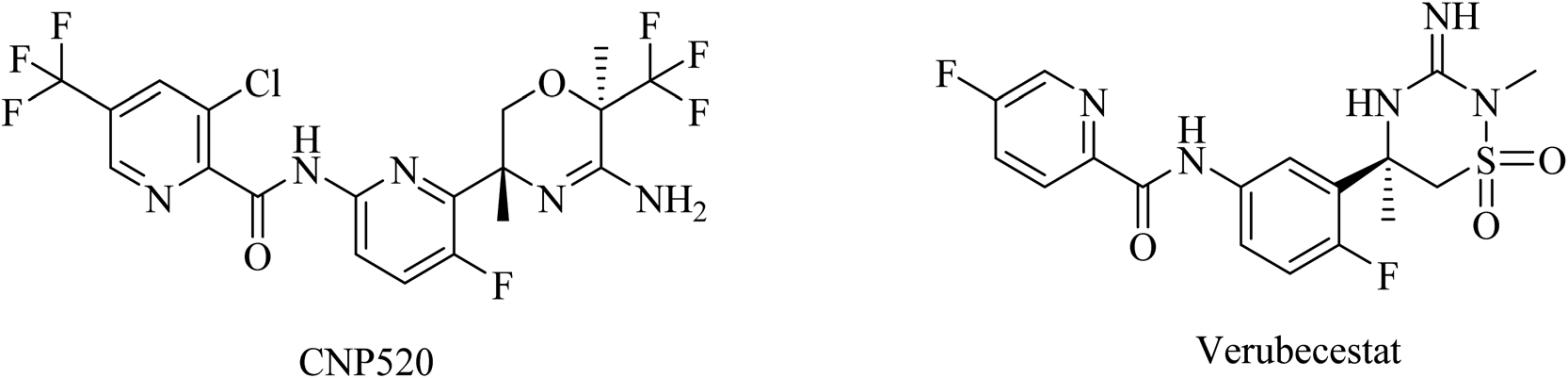
Some picolinamide based BACE1 inhibitors.

Umibecestat (CNP520) is another picolinamide-based (Fig. 16) oral BACE1 inhibitor.^155^ CNP520 was designed to meet the requirements of prevention treatment. In the preclinical studies, CNP520 revealed a considerable reduction in acute and chronic Aβ along with an acceptable safety profile.^156^ In 2019, CNP520 passed the phase II clinical trial, showing promising efficacy and tolerability. But it failed in phase III as it caused cognitive worsening in the treatment groups.^157^ The Alzheimer's Prevention Initiative Generation Program (Generation Study 1) announced that CNP520 worsens cognitive functions.^155^

Another picolinamide-based (Fig. 16) oral BACE-1 inhibitor is verubecestat (MK8931), which has been reported to reduce Aβ levels in AD patients.^158,159^ Nevertheless, in clinical trials, it caused no improvement in cognitive function in mild to moderate AD patients.^158^ Furthermore, some studies reported a greater decline in cognitive functions among patients receiving verubecestat than those receiving placebo.^160^

**Fig. 17:**
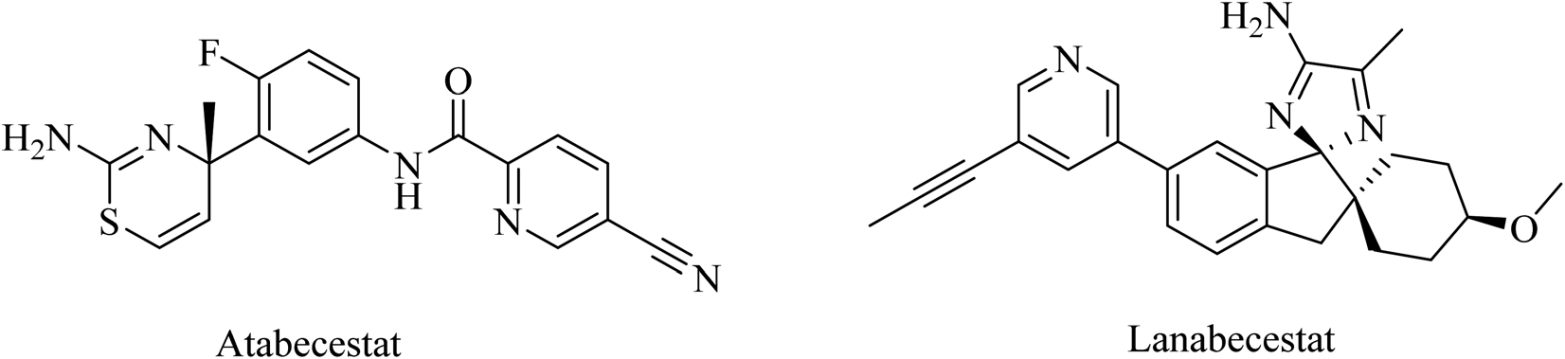
The chemical structure of some BACE1 inhibitors.

Atabecestat (JNJ-54861911), a picolinamide derivative (Fig. 17), was designed by Pharmaceutical Janssen as an oral BACE-1 inhibitor. In 2013, some subsequent clinical phase I studies of atabecestat have been conducted. It was launched in Belgium with a single increasing dose model, followed by a second study in addition to a similar trial performed in Japan. These studies were conducted on a limited number of healthy elderly volunteers and concluded promising results for atabecestat, which reduced Aβ aggregation after single or multiple doses.^161–163^ A subsequent phase IIb/III clinical trial (NCT02569398) was conducted to investigate the efficiency and safety of the drug, but it was terminated in 2018 because of the observed adverse effects, which include hepatotoxicity.^164,165^ In addition, other adverse effects on cognition, sleep, depression, and anxiety were reported for atabecestat in some other studies.^164,166^

Lanabecestat (Fig. 17) was developed as an anti-alzheimer drug that has the ability to pass the BBB and improve the clinical features, along with preventing the progression of the disease through inhibition of BACE1.^167^ The data obtained from some studies reflected adverse effects that include psychiatric disorders, weight loss, and change in hair color, while there was no considerable improvement in primary or secondary efficacy measures.^167,168^

**Fig. 18:**
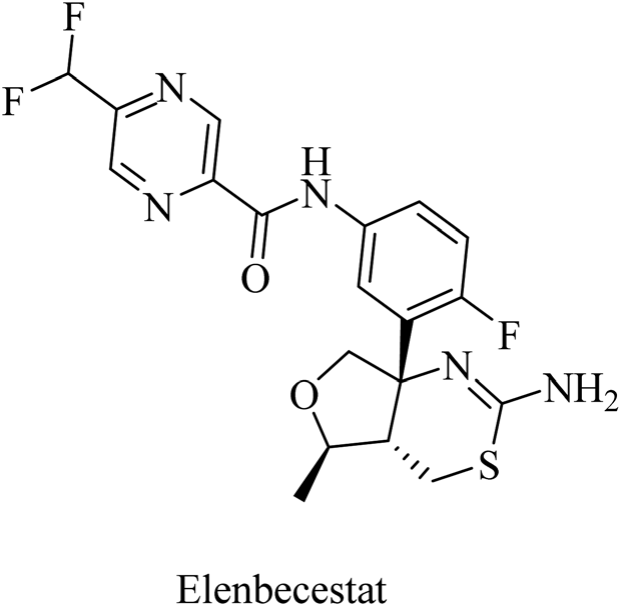
The chemical structure of elenbecestat.

Elenbecestat (E2609) is a pyrazinecarboxamide-based (Fig. 18) BACE1 inhibitor. It showed a dose-dependent reduction in the levels of Aβ in CSF. However, it showed no difference from placebo with regard to some other AD-related measures, such as the Alzheimer's Disease Composite Score (ADCOMS, *p* = 0.38) and CDR-SB (*p* = 0.55). Two 24 months studies of elenbecestat in a large number of mild AD patients with positive amyloid pathology biomarkers were discontinued in the sense that the toxicity induced outweighed the benefits obtained.^169^

In general, and on the basis of the published data relating to the effects of the oral BACE1 inhibitors in transgenic mouse models of AD, it can be concluded that BACE1 inhibitors are able to reduce the levels of Aβ in brain and CSF in a dose-dependent manner, but there is no solid evidence that they can improve cognitive functions effectively. In addition to the unfavorable toxicities observed in such studies and linked to BACE1 inhibitors, seventeen BACE1 inhibitors have failed in clinical trials to show considerable improvements in AD patients. Many of these trials were discontinued due to toxicity and/or cognitive worsening.^170^

#### 2.6.2. Dual BACE-1 and BACE-2 inhibitors

**Fig. 19:**
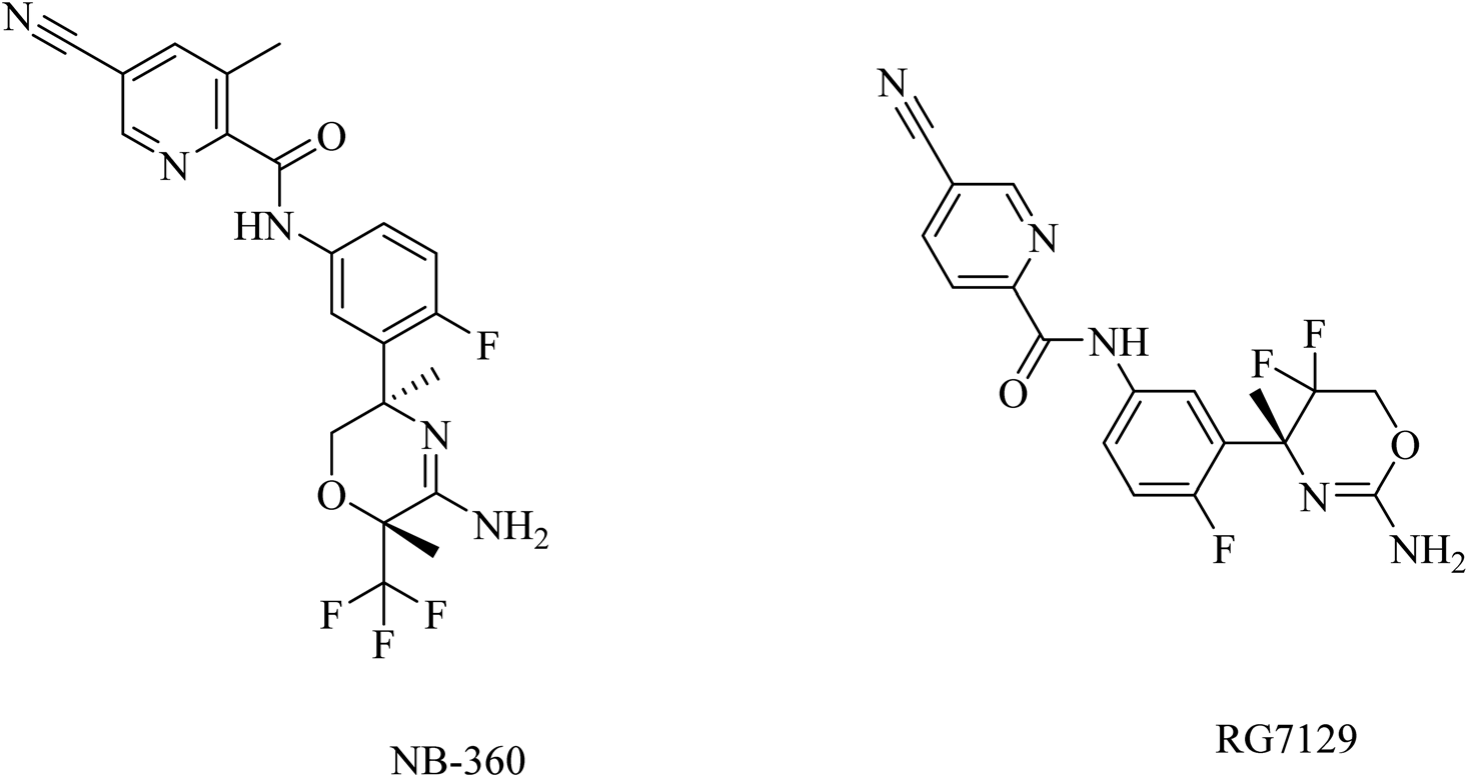
Picolinamide based inhibitors for both BACE1 and BACE2.

NB-360 was developed by Novartis Pharmaceuticals Corporation as a picolinamide derivative (Fig. 19) with potent BACE-1 and BACE-2 inhibition properties. It demonstrated an IC_50_ of 5.0 nM and 6.0 nM, against the two enzymes, respectively.^171^ In preclinical studies, NB-360 significantly inhibited the accumulation of Aβ in APP transgenic mouse brains. Similar data were obtained from clinical studies in rats and dogs. It also showed good BBB permeability.^172,173^ However, the safety profile of NB-360 was found disappointing. In some studies, it caused a hypopigmentation phenotype, which was attributed to the inhibition of BACE-2, which plays a crucial role in melanogenesis. Due to the effects of NB-360 on melanosome maturation and triggering hair depigmentation in a mouse model,^173^ studies on NB-360 were discontinued prior to clinical trials.^171^

RG712 (Fig. 19) was generated by Roche as a substituted phenylpicolinamide derivative carrying an oxazoline moiety. It was developed as an oral BACE inhibitor, and it showed an IC_50_ of 30 nM in the preclinical studies with minor selectivity over BACE2.^174^ It was evaluated in combination with an anti-Aβ antibody (gantenerumab) in some studies that involved AD transgenic mouse models, and it was found to reduce the amyloid plaques with slowing down the disease progression.^174^ However, the hepatotoxicity induced by RG712 prevented the completion of any further related clinical trials conducted by Roch.^175^

### 2.7. γ-Secretase acting drugs

In addition to the role of γ-secretase in the generation of Aβ from APP, it also proteolyzes many other type I integral membrane proteins, in particular the Notch receptor, which plays a role in many essential steps during cell differentiation.^176^ So targeting γ-secretase as a significant approach to the treatment of AD should avoid affecting Notch signaling in order to show a good safety profile.^177^

#### 2.7.1. γ-Secretase inhibitors (GSIs)

**Fig. 20:**
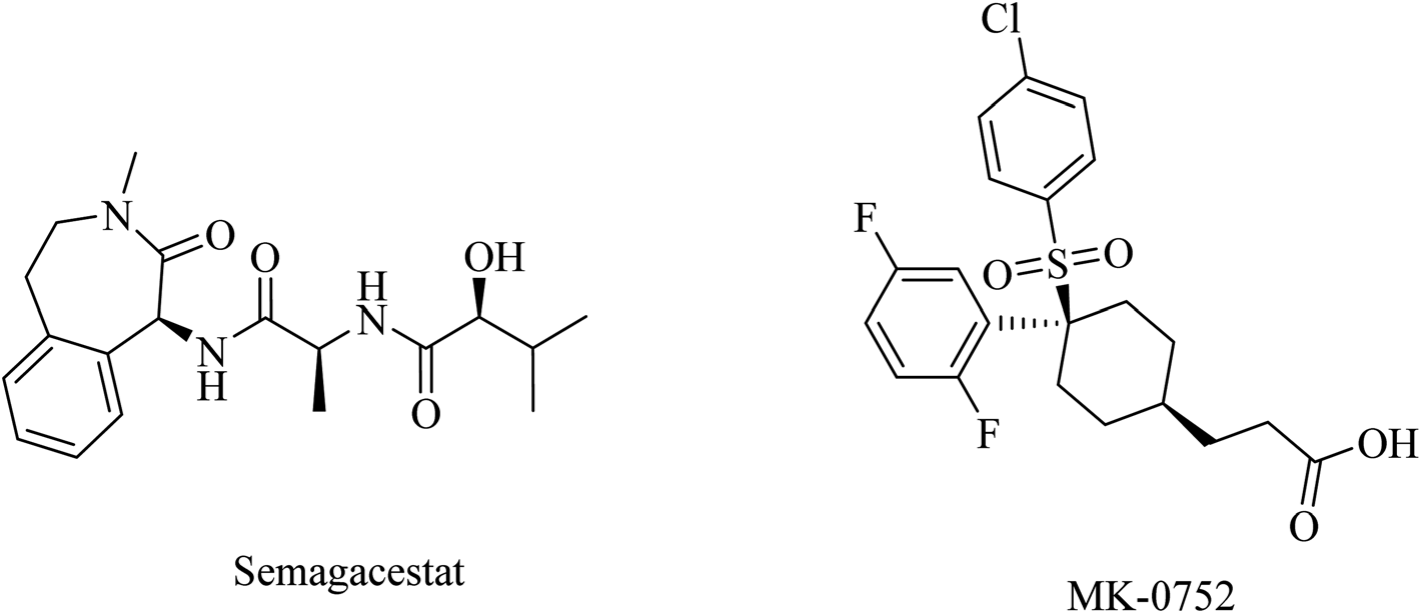
The chemical structure of some γ-secretase inhibitors.

Semagacestat (LY450139) (Fig. 20) is a benzoazepine-based γ-secretase inhibitor developed by the Eli Lilly pharmaceutical company.^178^ Semagacestat decreases in a dose-dependent manner brain, CSF, and plasma Aβ in animals as well as CSF and plasma Aβ in humans in comparison to placebo-treated patients.^179^ However, in a phase III clinical study, semagacestat did not improve cognitive functions and triggered, in high doses, adverse effects such as worsening functional abilities, infections, and skin cancers.^180^

MK-0752 (Fig. 20) is another GSI that does not differentiate between γ-secretase and Notch receptor.^154^ In a phase I clinical trial, evaluation of its safety, pharmacokinetics, and pharmacodynamics on the basis of a single oral dose has been conducted in 27 healthy volunteers.^181^ The data obtained revealed that MK-0752 is safe and reaches its maximum plasma concentration in approximately 3–4 h with a *t*_1/2_ of 20 h as well as greatly reducing Aβ1-40 levels in CSF for 12 h.^154^ However, the safety profile was disappointing in a phase II study because it did not differentiate between γ-secretase and Notch.^154^

**Fig. 21:**
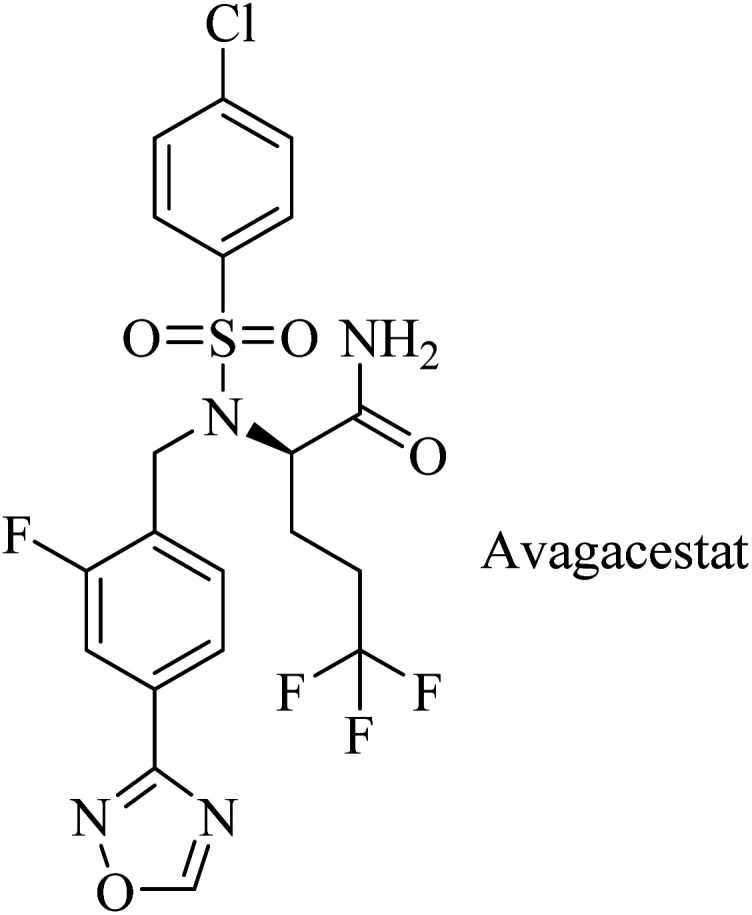
Benzenesulfonamide based GSI.

Avagacestat (BMS-708163) is a benzenesulfonamide-based (Fig. 21) potent and selective GSI developed by Bristol-Myers Squibb.^182^ It revealed a 193-fold selectivity for APP over Notch blockade. It inhibited Aβ40 production with an IC_50_ of 0.30 nM, resulting in lowering the levels of Aβ40 in CSF, brain, and plasma as studied in dogs and rats.^183^ Phase I study demonstrated that the safety, tolerability, pharmacokinetics, and pharmacodynamics properties of BMS-708163 were promising after oral administration in healthy, young volunteers (NCT01454115). Accordingly, it was decided to be evaluated by further clinical trials. In phase II trials, BMS-708163 was evaluated in 209 outpatients with a median age of 75 years, diagnosed with mild-to-moderate AD. Different doses were examined in this study, and the results revealed that it cannot be tolerated at a dose of 100 mg or above. Additionally, a worsening of cognition was observed at such doses. Meanwhile, the dose up to 50 mg day^−1^ showed results similar to those of placebo.^184^ The obtained results have been confirmed by a subsequent study conducted on CSF biomarker-negative volunteers, which demonstrated deterioration in the health conditions of patients with the occurrence of nausea, vomiting, diarrhea, rash, and nonmelanoma skin cancers. Meanwhile, there were no therapeutic benefits to the lower doses.^185^

**Fig. 22:**
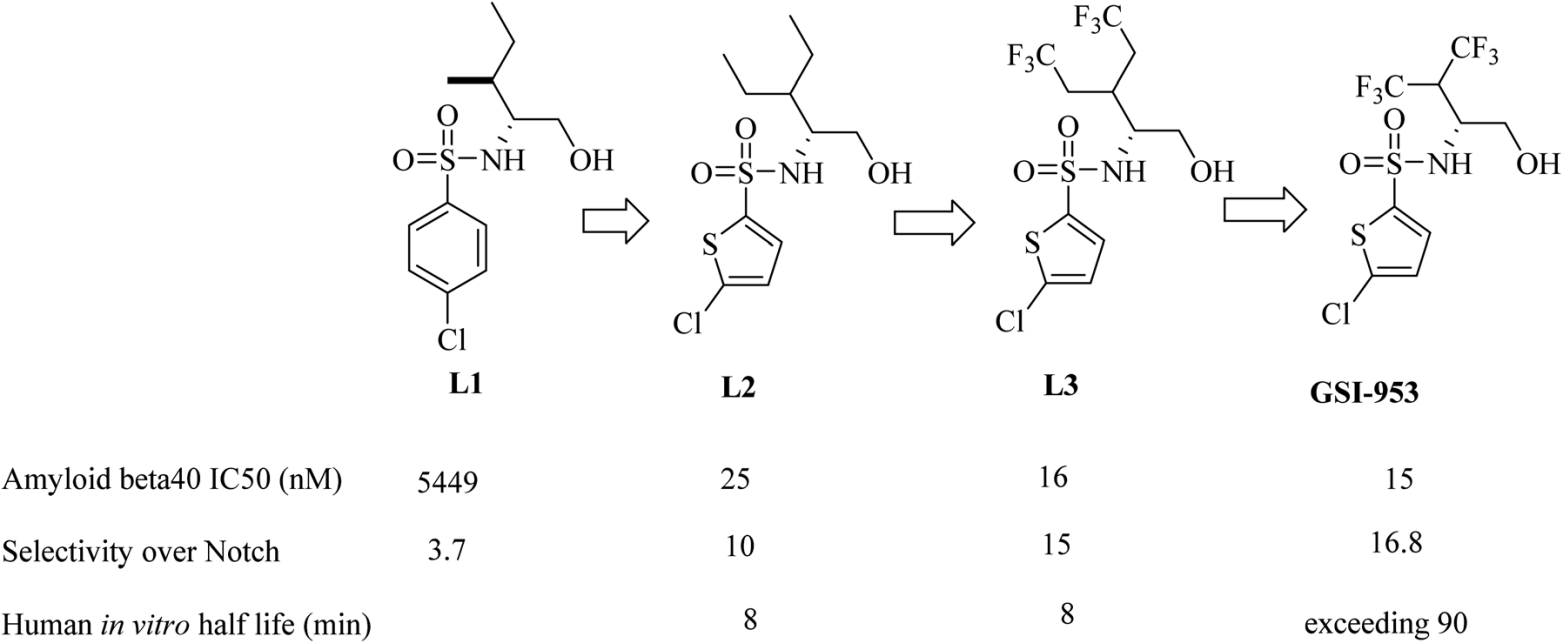
Lead optimization in the development of GSI-953.

Begacestat (GSI-953), 2,5-disubstituted thiophene sulfonamide (Fig. 22), is considered a second generation GSI. It was generated by Wyeth (now Pfizer).^186^ Researchers at Wyeth applied the high throughput screening technique to get the molecule LI (Fig. 22).^187^ L1 was then modified to afford the more optimized L2, which in turn led to the discovery of L3, which showed much potent inhibition for Aβ production,^183^ as can be seen in Fig. 22. The most important character of L2 and L3 is that they revealed much better selectivity for APP over Notch. But unfortunately, they were metabolically unstable, and this was attributed to the easy oxidation of the methylene group. So, the alkyl side chain was contracted to afford a series of new molecules, from which GSI-953 was discovered (Fig. 22). GSI-953 was found to be relatively more resistant to rapid metabolism, showing a half-life of more than 90 min. Meanwhile, GSI-953 displayed comparatively better properties with respect to inhibition of Aβ production and selectivity for APP over Notch, as presented in Fig. 22.^183,186^ GSI-953 revealed promising data in phase I clinical trials as it inhibited Aβ production at nanomolar concentrations, and showed 16-fold selectivity for APP over Notch. In a study conducted on a human APP-overexpressing Tg2576 transgenic mouse model, oral use of GSI-953 led to a significant reduction in Aβ levels in CNS, brain, and plasma. Similar results have been obtained when the effects of oral use of GSI-953 were evaluated in healthy human volunteers.^188^

**Fig. 23:**
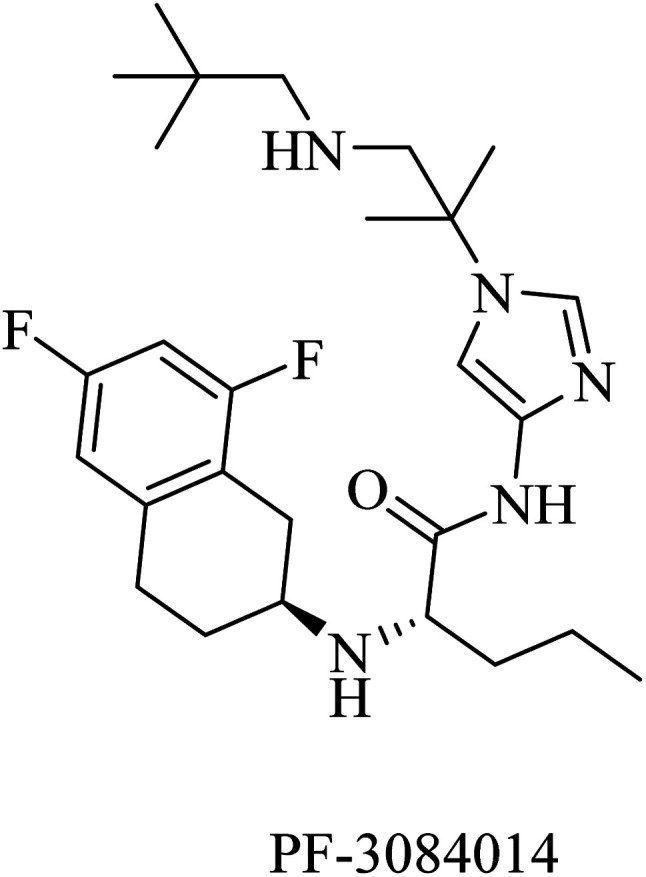
Notch sparing GSI.

PF-3084014 is an imidazolylpentamide-based Notch-sparing GSI (Fig. 23). It was reported as a non-competitive reversible human GSI with an IC_50_ of 1.3 nM.^189,190^ With respect to selectivity, PF-3084014 displayed minor inhibition of Notch signaling (IC_50_ = 19.15 nM). The selectivity index of PF-3084014 was found to be 1473 for APP over Notch. So it was considered a Notch sparing GSI. It showed good penetration to the BBB with a reduction in Aβ levels in animals. However, there is a lack of data about its effect on amyloid plaque deposition in transgenic mice, as well as no data available about its behavioral effects in AD animal models.^154^

In clinical trials, GSIs such as semagacestat and avagacestat reduced Aβ production in AD patients.^184,191^ However, the multiple effects of such inhibitors prevented further progress in the clinical trials due to inhibition of Notch signaling,^192^ which induces adverse effects such as gastrointestinal disturbance, infection, worsening cognition, and the risk of skin cancer.^193^ To address this problem, a new class of γ-secretase acting molecules that specifically regulate or modulate this enzyme are required.^193^

#### 2.7.2. γ-Secretase modulators (GSMs)

This class of drugs have the ability to modulate the activity of γ-secretase by regulating specific activities of the enzyme rather than the whole inhibition of the enzyme. GSMs are far more interesting disease-modifying agents than GSIs because GSMs (1) selectively inhibit Aβ42 production and aggregation; (2) increase the production of shorter Aβ37 or Aβ38 rather than Aβ42; (3) do not affect the total Aβ production and the accumulation of APP-CTF; and (4), most importantly, spare Notch signaling.^194^

##### 2.7.2.1. First-generation GSMs

**Fig. 24:**
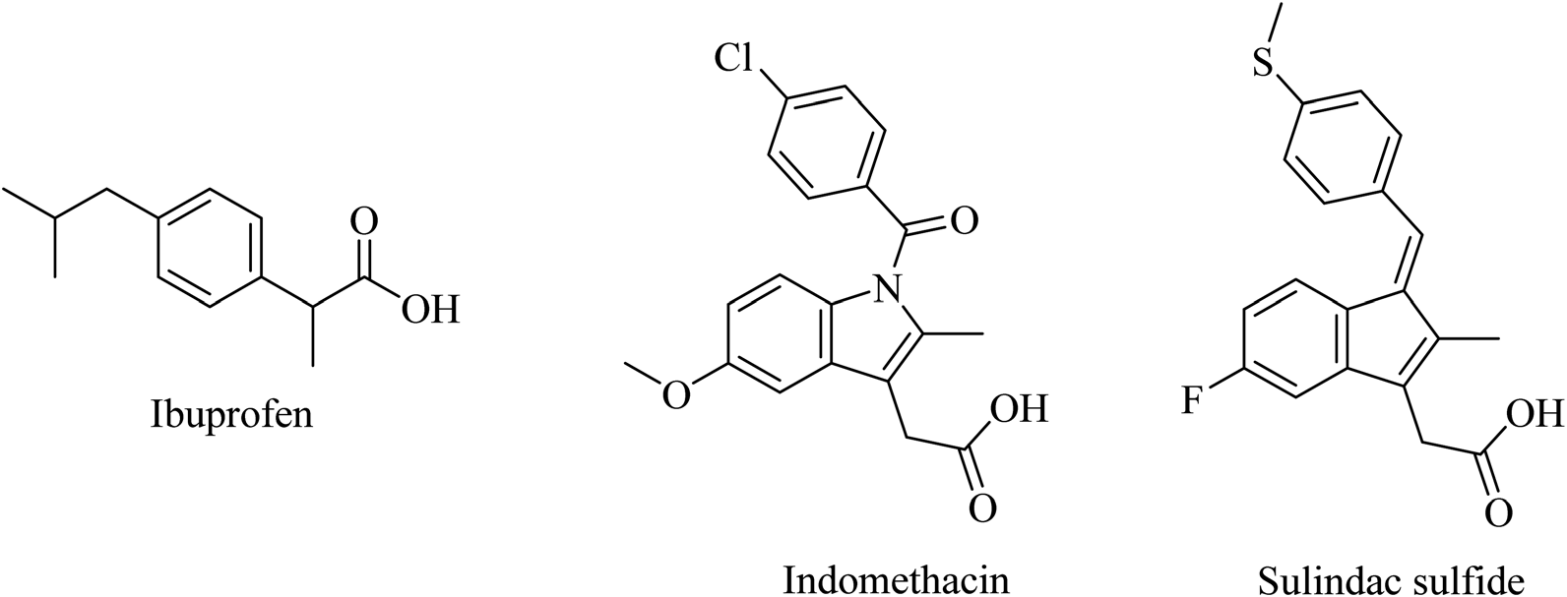
First generation of NSAIDs as GSMs.

Nonsteroidal anti-inflammatory drugs (NSAIDs) represented the first generation of GSI as they were found to modulate the activity of γ-secretase in a manner that reduces Aβ42 and increases the soluble Aβ38 without inhibiting Notch. Ibuprofen, indomethacin, and sulindac sulfide (Fig. 24) are examples of this class of NSAIDs.^194,195^

Unfortunately, ibuprofen, indomethacin, and sulindac sulfide lack good brain penetration, so they showed insignificant data in clinical trials associated with AD models.^196^

##### 2.7.2.2. Second-generation GSMs

The poor brain penetration of NSAIDs was required to be resolved in the second-generation GSMs, which can be classified into different categories, including NSAID-derived carboxylic acid GSMs, non-NSAID-derived imidazole GSMs, and natural product-derived GSMs.^194^

##### 2.7.2.3. NSAID-derived carboxylic acid GSMs

**Fig. 25:**
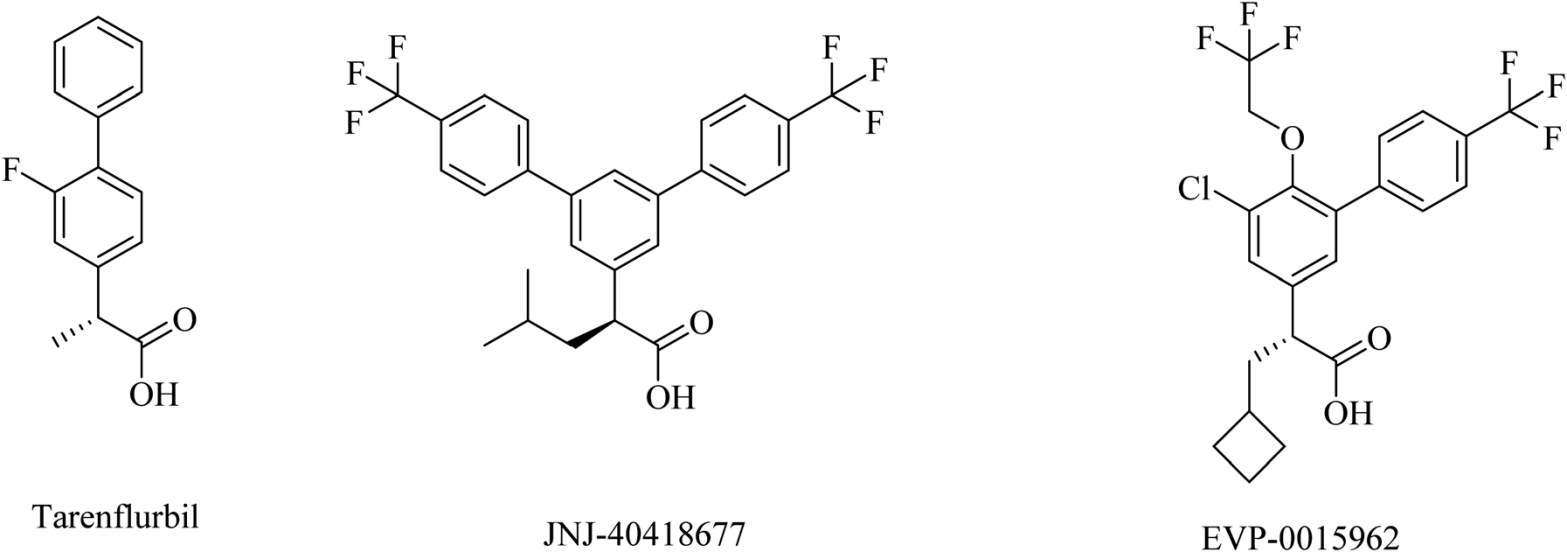
NSAID derived carboxylic acid GSMs.

Tarenflurbil ((*R*)-flurbiprofen), an aryl propionic acid derivative (Fig. 25), was strongly suggested as a clinical candidate for AD treatment.^197^ It showed no Notch inhibition-related adverse effects;^198^ however, it still showed insufficient brain penetration and minor efficacy, leading to its failure in phase III clinical study.^199–201^

JNJ-40418677 was generated by Janssen as an analog of flurbiprofen by addition of substituents on the core aryl ring (Fig. 25). JNJ-40418677 has sufficient lipophilicity to penetrate the brain and reveal its therapeutic activities. It was found to reduce Aβ42 levels and elevate the levels of Aβ38 in the brain without affecting the levels of total Aβ in the brain. The safety profile of JNJ-40418677 still needs to be evaluated.^202^

EVP-0015962 is a cyclobutyl group containing analog of (*R*)-flurbiprofen (Fig. 25), it was generated by Forum Pharmaceuticals. It displayed good data, such as a considerable reduction in Aβ42 levels when administered orally with good efficacy in animals.^203^ Nevertheless, the high lipophilicity was considered an unfavorable property of this drug.^196^ So its clinical trials were discontinued after phase II.^204^

**Fig. 26:**
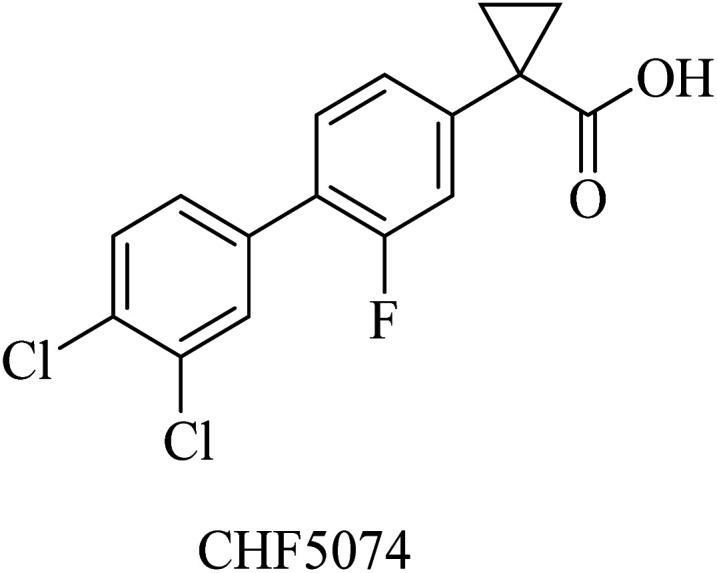
The chemical structure of itanapraced.

Itanapraced (CHF5074) is another carboxylic acid derivative (Fig. 26) designed by Chiesi Pharmaceuticals as a GSM. It was found to enhance memory and reduce microglial activation in Tg2576 mice.^205^ It did not show a reduction in Aβ42 levels either in plasma or CSF; instead, it decreased the levels of CD40, which is considered a marker for microglia activation. So CHF5074 is now classified as a microglia modulator.^196,206^

**Fig. 27:**
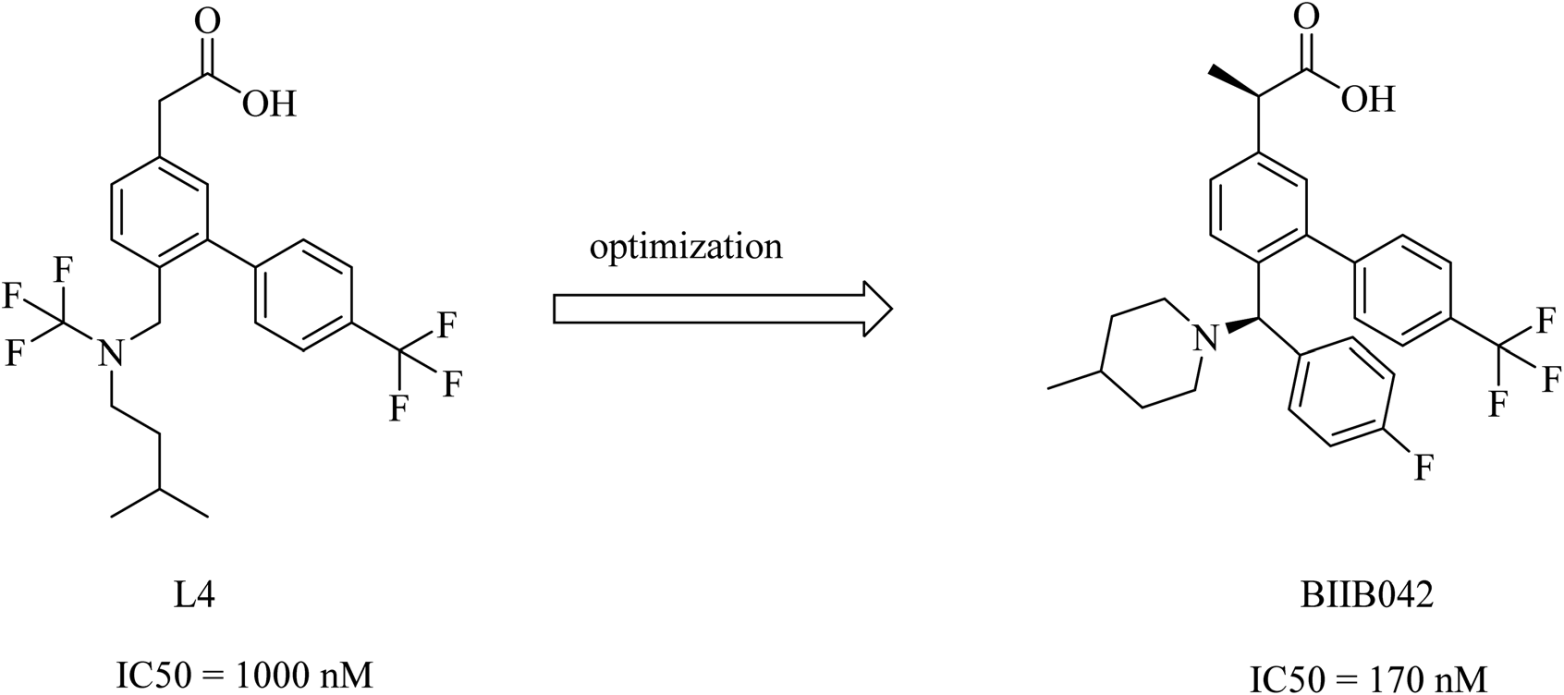
Biogen's NSAID-derived carboxylic acid GSM 1 and an optimized modulator BIIB042.

Biogen's research was focused on the discovery of NSAID derived carboxylic acid GSMs. In 2011, Biogen reported^203^ the discovery of a potential preclinical GSM candidate (BIIB042). Its development was based on the optimization of the previously examined L4, as shown in Fig. 27. This lead compound showed considerable potency, but it had poor brain penetration. So, the researchers worked on improving this criterion. More than one modification has been done on the basis of deduced SAR, which revealed that the activity was improved by the creation of a chiral center by adding a methyl group at the α position of the carboxyl group in addition to a rigid piperidinyl moiety, as can be noticed in Fig. 27. This optimization afforded the preclinical candidate BIIB042, which showed a reduction in Aβ42 levels with a concomitant elevation in Aβ38 levels and no effect on Aβ40 levels. Several clinical studies have been conducted by Biogen, which has published about 66 *in vivo* efficacy studies of BIIB042. The published data indicated that BIIB042 decreased Aβ42 levels and increased Aβ38 levels, but showed no effect on Aβ40 levels in the brains and plasma of mice and rats. These pharmacodynamic properties have been confirmed by similar results concluded from a study conducted on monkeys administered orally a single dose of BIIB042.^203^

In addition to these promising data, BIIB042 did not inhibit Notch signaling in an *in vitro* model.^207^ In a more advanced study conducted on human APP-overexpressing Tg2576 mice, BIIB042 reduced the levels of Aβ42 and decreased the amyloid plaque burden.^208^ BIIB042 is a candidate drug for AD.^209^

##### 2.7.2.4. Non NSAID imidazole GSMs

**Fig. 28:**
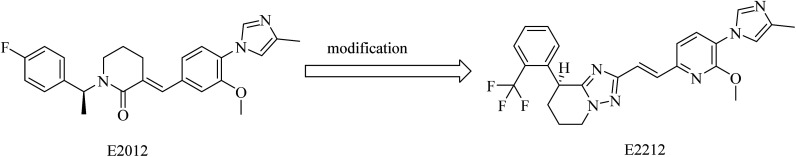
Non NSAID imidazole GSMs: E2012 and E2212.

This class of compounds attracted the attention of researchers of Eisai group as they focused on the development of non-carboxylic acid GSMs. At first, nearly all non-carboxylic acid GSMs had a general structural feature of an arylimidazole moiety linked *via* an olefin to a lactam, as in E2012, or a heterocycle, as in E2212 (Fig. 28). They developed E2012 as a substantial GSM with an IC_50_ of 83 nM and it was the first non-carboxylic acid molecule to be studied clinically as a GSM.^154^ The clinical trials revealed a question concerning the safety of E2012, which induced cataracts in rats.^210^ As a consequence, its clinical trials have been terminated and exposed to a series of modifications by Eisai, which finally developed E2212 (Fig. 28).^211^

It was reported that E2012 was modified to E2212 for improving the drug-likeness.^154^ E2212, in its phase I clinical trials, showed comparatively better safety in comparison to E2012.^198^ Diarrhea was the most observed adverse effect for E2212.^198^ It also revealed promising efficacy by reducing the levels of Aβ42 by 54%,^212^ showing an IC50 of 17 nM.^213^ Although the structure of E2212 has never been revealed, it is predicted by several Eisai process chemistry patents. The further development of this compound has not been reported.^154^

**Fig. 29:**
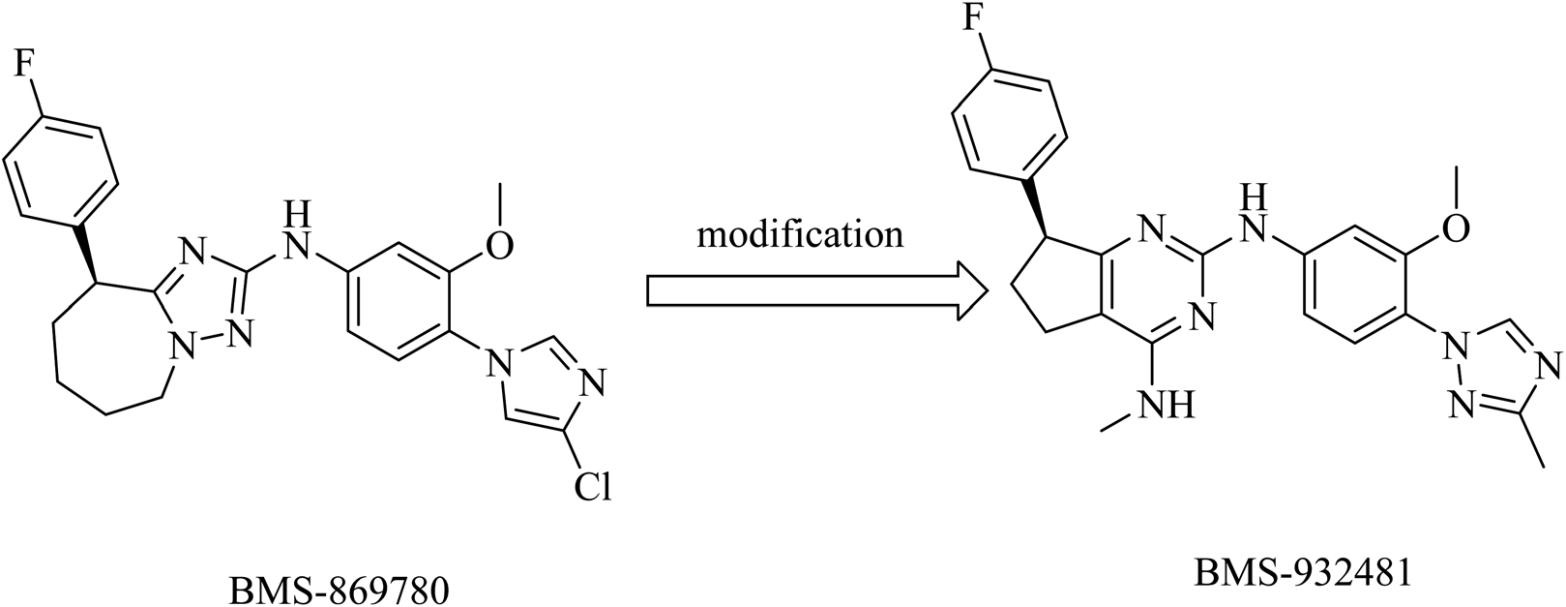
Non NSAID derived GSMs.

Compound BMS-869780 (Fig. 29) was developed by Bristol-Myers Squibb as a non-NSAID imidazole GSM. It showed promising activity in mouse and rat brains by a significant reduction of Aβ42 levels after oral administration.^214^ Although there was no evidence that BMS-869780 triggers side effects related to Notch inhibition, further clinical studies have been terminated because of a potential toxicity issue, as the daily dose required to provide therapeutic effects in human AD was very high (700 mg). BMS-869780 was then modified to BMS-932481 (IC_50_ of 7 nM) in order to improve the pharmacodynamics, as shown in Fig. 29. It can be noticed that the chloroimidazole nucleus was replaced by the methyltriazole moiety.

BMS-932481 was evaluated for pharmacokinetics, which appeared to be promising. However, further studies have been terminated due to safety issues.^154^

**Fig. 30:**
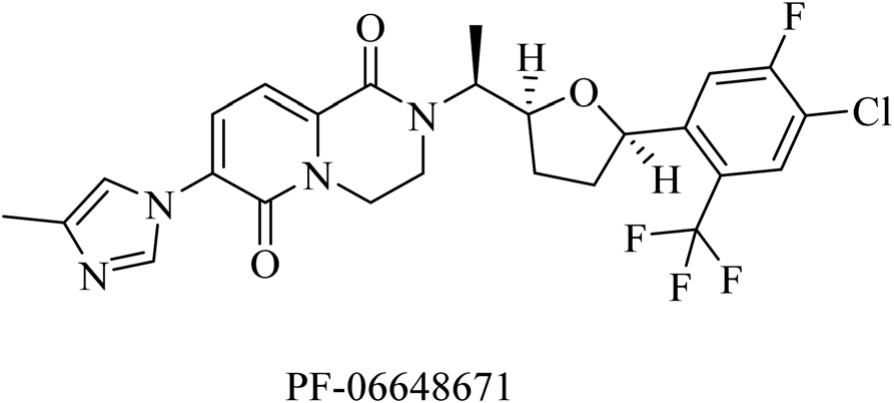
The chemical structure of the non NSAID imidazole GSM developed by Pfizer.

PF-06648671 (Fig. 30) was developed by Pfizer, and it was found to be of good tolerability at single doses given to healthy persons. It decreased the plasma levels of Aβ40 and Aβ42. Meanwhile, it increased the levels of Aβ37 and Aβ38.^215^ However, it was discontinued due to Pfizer's discontinuation of R&D in neurology in 2018.

**Fig. 31:**
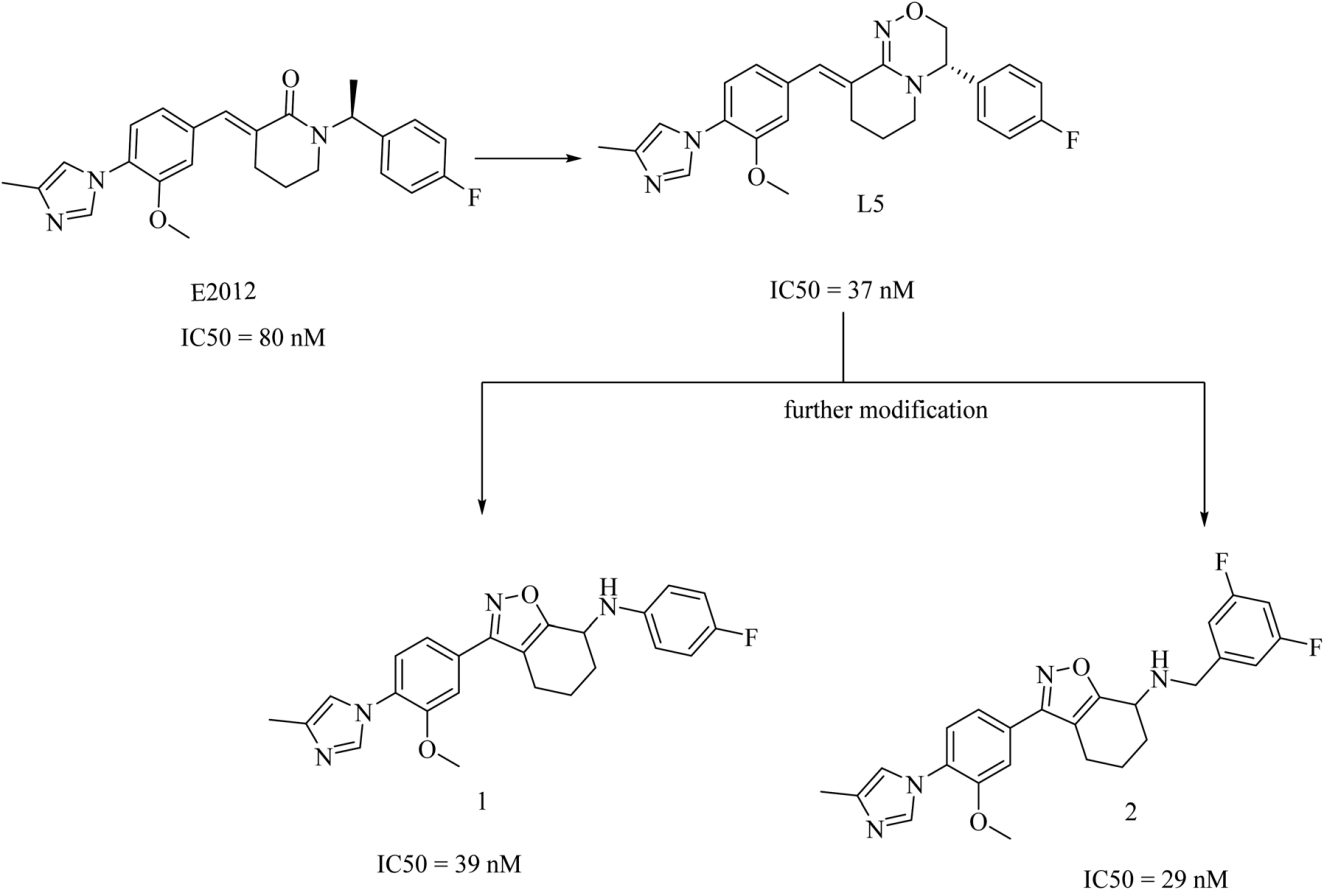
Merck Research Laboratory's oxadiazine L5 and tetrahydrobenzisoxazole analogs 1 and 2.

In 2017, researchers at Merck reported the discovery of a new series of 17 tetrahydroisoxazole molecules as GSMs developed on the basis of Eisai's E2012 (ref. 216) and their own preceding GSM molecules, among which compounds is L5 (ref. 196) (Fig. 31). The double bond and the lactam carbonyl in Eisai's E2012 are sites of potential metabolism, so they were replaced by a tetrahydrobenzisoxazole moiety, as shown in Fig. 31. Another benefit of the tetrahydrobenzisoxazole group is avoiding the stereochemistry of the double bond. The synthetic compounds showed variable activities, from which SAR has been deduced to develop some compounds with good selectivity and potency. The most promising candidates were compounds 1 and 2, showing an IC_50_ of 39 nM and 29 nM against Aβ42, respectively (Fig. 31). It can be noticed that 1 and 2 were far more potent than E2012 and L5. The promising criteria of 1 and 2 encouraged their evaluation in a preclinical rat model. Compound 1 reduced CSF Aβ42 levels by 58%, compared to only a 20% reduction in Aβ levels reported for compound 2 after 3 h of a single oral dose in rats. Interestingly, compound 2 was more potent than 1*in vitro*. To find an explanation for this controversy, the levels of both drugs in the plasma and brain of rats have been assessed. The concentration of compound 1 in the brain was found to be sixfold that of compound 2, which gave an account of the better results of compound 1 in animal model study. Meanwhile, the efflux ratio of 2 was four-fold higher than that of 1. Despite these promising data, we did not find any further data concerning the safety and efficacy of compound 1 or its future evaluation in clinical trials.^196^

##### 2.7.2.5. Natural products as gamma secretase modulators

**Fig. 32:**
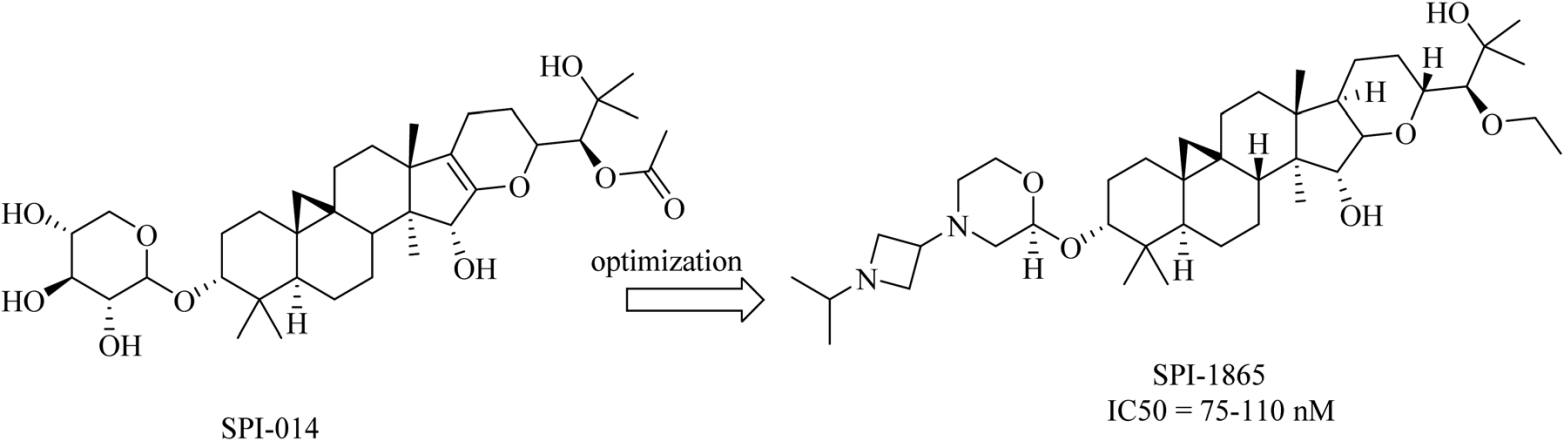
Natural and semisynthetic analogs as GSMs.

Compound SPI-014 (Fig. 32), isolated from the extract of *Actaea racemosa* (black cohosh), was found to exhibit activities like GSMs. Several modifications have been done on SPI-014, and a lot of semisynthetic analogs have been developed. The modifications included the substitution of both sugar and acetate moieties with more stable groups to enhance drug-like properties, as can be seen in Fig. 32. These modifications led to the discovery of a new drug molecule, SPI-1865 (Fig. 32). Despite the improvement in the pharmacodynamics of the modified molecule, it did not pass the clinical trials due to the unanticipated off-target adrenal toxicity reported.^217^

**Fig. 33:**
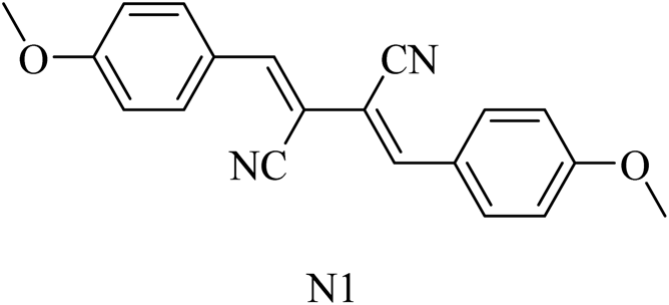
Natural GSM.

2,3-Bis((*Z*)-4-methoxybenzylidene)succinonitrile (N1) (Fig. 33) was extracted from the marine sponge-derived fungus *Dichotomomyces cejpii*. It was found to decline the excessive production of Aβ42 in an AD cellular assay.^218^

**Fig. 34:**
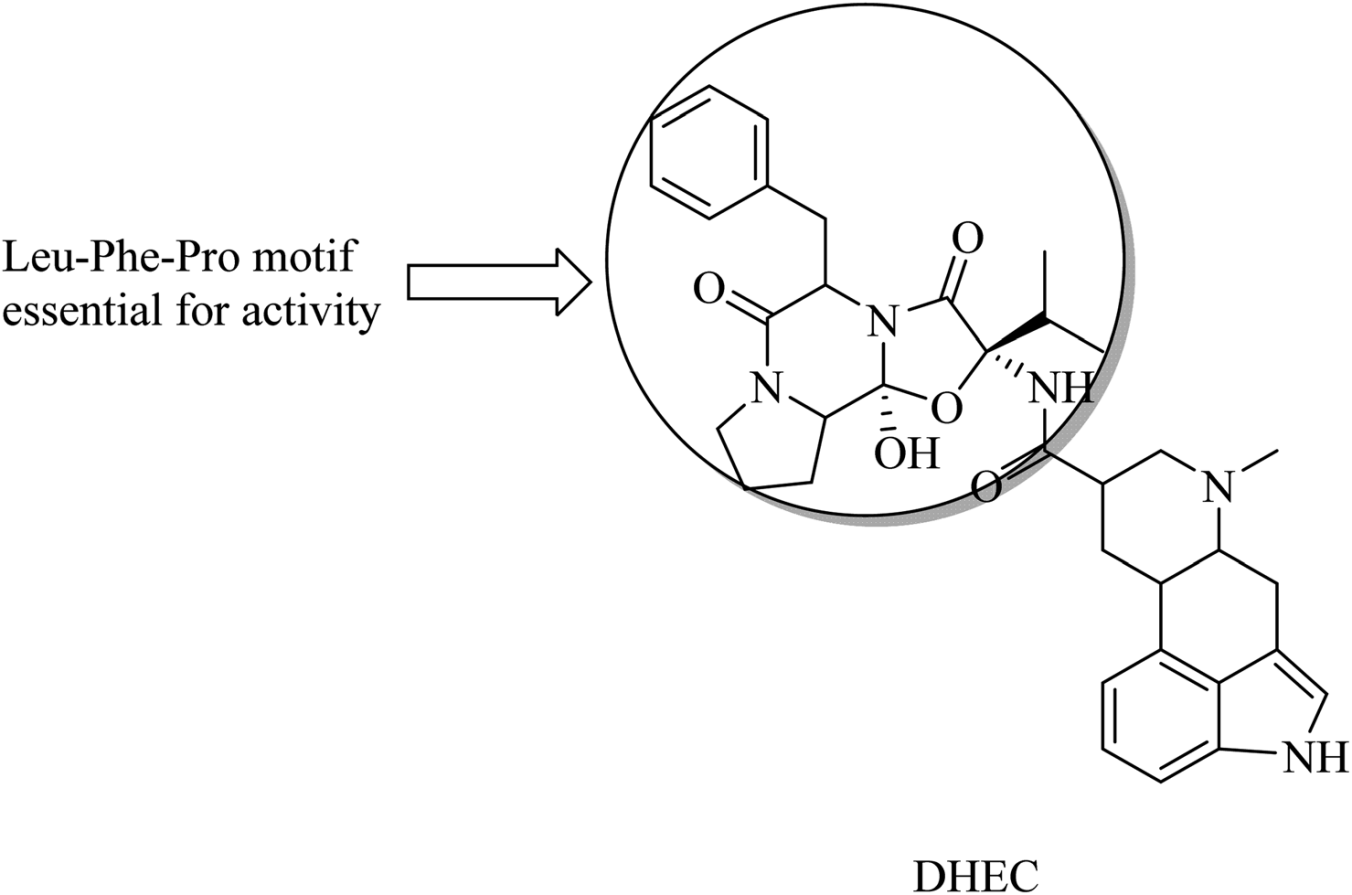
The chemical structure of DHEC.

Dihydroergocristine (DHEC) (Fig. 34) is an FDA-approved natural drug that revealed significant data with respect to selective inhibition of APP. It has a Leu-Phe-Pro motif, which plays a crucial role in binding to the allosteric site of the enzyme. This allosteric site represents the region to which APP-C99 interacts to be cleaved. So DHEC competes with APP for binding to γ-secretase, reducing the production of Aβ42 in the brain.^219^ DHEC was found to reduce the levels of Aβ at micromolar concentrations when examined *in vivo*.^219^

### 2.8. α-Secretase activators

Activation of α-secretase was suggested as a potential therapeutic strategy in order to inhibit the aggregation of amyloid plaques.^220^ This is because of the role of α-secretase in enhancing the proteolysis of APP in the non-amyloidogenic pathway and, hence, decreasing the formation of amyloid plaques. A series of membrane-bound proteases (a disintegrin and metalloprotease family) regulate the α-secretase.^221^ ADAM10, ADAM17, and ADAM9 have been suggested as α-secretases.^222^

**Fig. 35:**
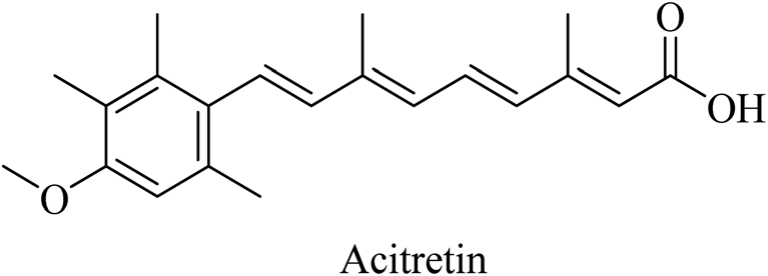
The chemical structure of acitretin.

Synthetic retinoids were proven to improve the nonamyloidogenic proteolysis of APP. One of the important synthetic retinoids in this regard is acitretin, a vitamin A analog (Fig. 35). Acitretin was evaluated in a phase II clinical study and was found to increase ADAM10 expression as well as reduce the levels of Aβ in APP/PS-1 transgenic mice.^223^ Furthermore, it enhances the stimulation of the mature ADAM10, resulting in higher activity of α-secretase in neuroblastoma cells.^223^ One of the encouraging features of acitretin is its ability to cross the BBB easily, and its level is not affected by glycoprotein (P-gp).^224^ On the other side, it was linked to some severe toxicity, such as alopecia, peeling, cheilitis, and hepatotoxicity.^154^

**Fig. 36:**
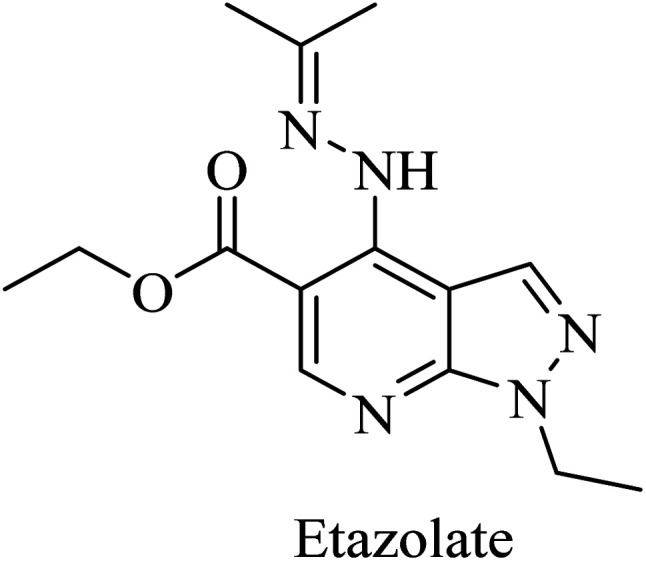
The chemical structure of etazolate.

Etazolate (EHT-0202), a pyrazolopyridine derivative (Fig. 36), is a gamma-aminobutyric acid-A (GABA_A_) receptor modulator. It was found to stimulate α-secretase and enhance sAPPα production.^225^ EHT-0202 has been assessed in a phase II clinical study in mild-to-moderate AD subjects. It showed cognitive improvement along with a good safety profile and tolerability.^225,226^

### 2.9. 5HT-receptors

One of the receptors that emerged lately as potential targets for cognitive disorders and AD is serotonin.^227,228^ In this regard, 5-HT6R and 5-HT7R are the most extensively studied serotonin receptors because of their high distribution in the brain and association with cognitive properties *in vivo*.^229^ Furthermore, 5-HT6R signaling was found to be associated with changes in cholinergic and glutamatergic functions in the brain, with little peripheral effect.^230^ However, the clinical trials against AD revealed no evidence for the therapeutic activity of any of the selective 5-HT6R or 5-HT7R drugs.^57^

**Fig. 37:**
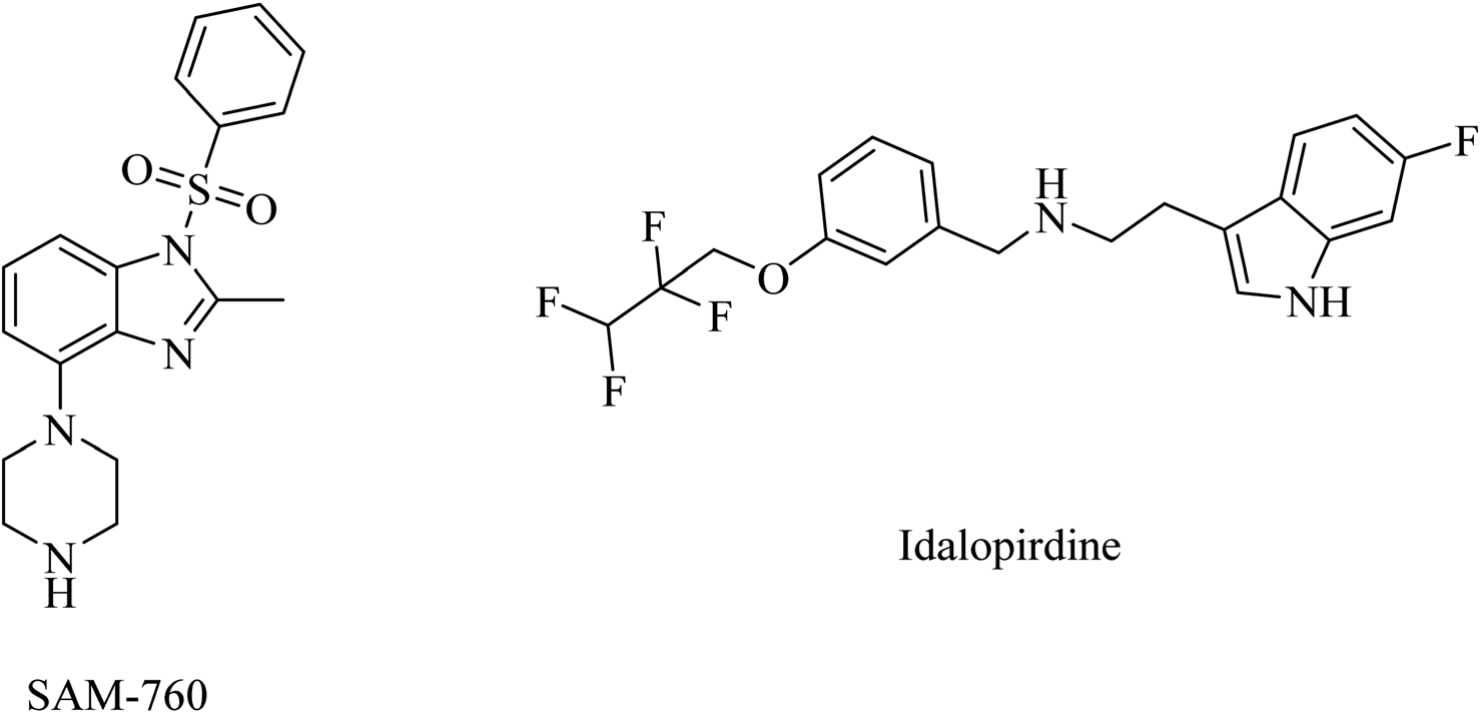
The chemical structures of selective 5-HT6R antagonists.

PF-05212377 (SAM-760), a benzimidazole derivative, and idalopirdine, an indole-based molecule, are selective 5-HT6R antagonists (Fig. 37).^57^ Clinical studies demonstrated that SAM-760 had no therapeutic benefits with regard to cognitive disorders; however, it showed good safety and tolerability.^57,231^

Similarly, idalopirdine was ineffective for AD patients, with a risk of elevated liver enzymes and vomiting.^232^ These data reveal the insignificance of idalopirdine for the treatment of AD.^233^

### 2.10. Glutaminyl cyclase (QC) inhibitors

The importance of QC in the development of anti-alzheimer drugs originated from the information that QC catalyzes the formation of cerebral pyroglutamate-Aβ 3 (AβpE3), which is considered one of the most neurotoxic forms of Aβ.^234,235^ So, inhibition of QC is suggested as a potential therapeutic approach to AD treatment.^234^ The work on the development of QC inhibitors has drawn attention in the last decade, where the design developed a zinc-binding group in order to coordinate the Zn^2+^ ion incorporated in the active site in addition to other common features.^235^ One of the outstanding works in this regard was done by the Probiodrug company, which rationally developed some promising QC inhibitors and identified the imidazole nucleus as a zinc chelating weak QC inhibitor.^235^

**Fig. 38:**
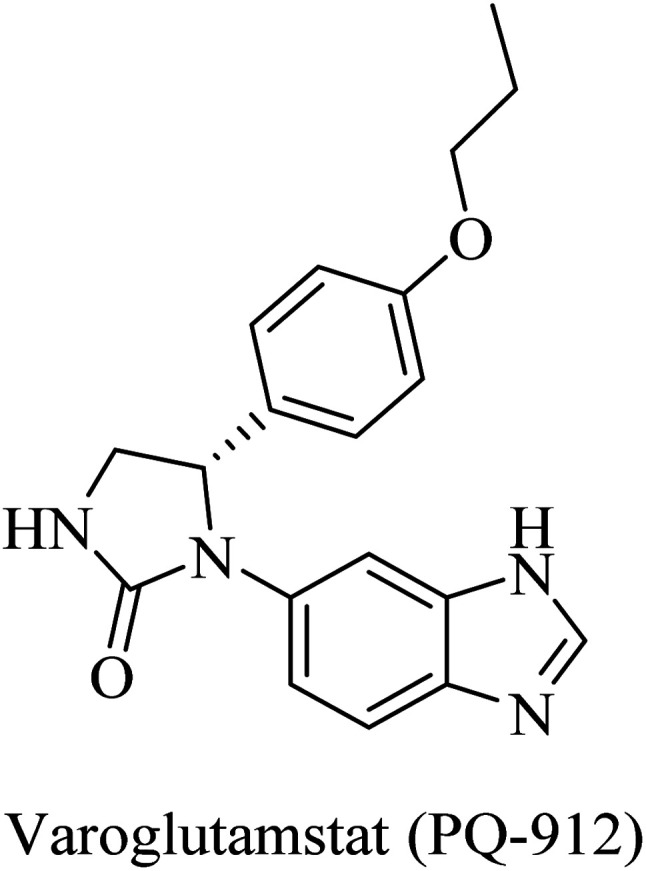
Benzimidazole based QC inhibitor.

PQ-912, a benzimidazole-based molecule (Fig. 38), showed competitive inhibition for QC with a Ki value of 25 nM. It was found to interact through coordination of the zinc ion in the QC's active site.^235^ PQ912 was evaluated for pharmacokinetic properties, which were found to be acceptable with good safety and tolerability in doses up to 200 mg.^236^ The studies have identified the maximum tolerated dose to support further studies at lower doses.^237^ Despite the promising cognitive improvement reported, many studies on PQ912 have been discontinued due to high dose toxicity.^238^

The results obtained from clinical evaluation of PQ912 have proven that QC is a significant AD druggable target. Furthermore, QC inhibition was found to improve synaptic functions by decreasing the toxic effects of AβpE3. In addition to this, long-term use is likely to modify the disease and reduce the neuroinflammations associated with AD.^234,235^

### 2.11. Anti-inflammatory agents

Chronic inflammation of the cerebral neurons is one of the pathological hallmarks linked to AD.^239^ It was suggested that the origin of neuroinflammation is the activation of glial cells by triggering factors such as neural environment or neuronal injury. One of the crucial factors in this scenario is TNF-α, which plays a pivotal role in neuronal excitotoxicity, neuroinflammation, and synapse loss. Another role for TNF-α associated directly with AD pathogenesis is enhancing amyloidogenesis and upregulating BACE-1 expression.^240–242^

Etanercept is a competitive TNF-α inhibitor. It is a fully human dimeric fusion protein consisting of the extracellular ligand-binding domain of the human 75 kilodalton TNF-α receptor linked to the Fc portion of human immunoglobulin G1 (IgG1).^243^ Clinical studies revealed that it had no therapeutic benefits in clinical studies against alzheimer.^243^ Etanercept limitation is related to its pharmacokinetics; it is unable to cross the brain–blood barrier.^243^

**Fig. 39:**
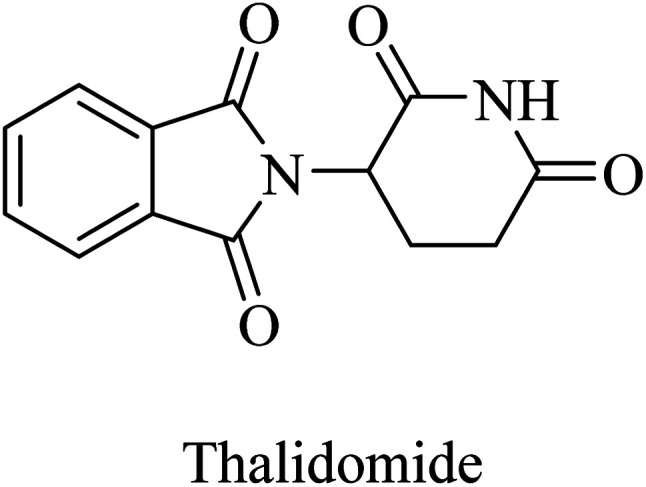
The chemical structure of thalidomide.

Thalidomide, another TNF-α inhibitor, is a small molecule composed of a phthalimide nucleus attached to a glutarimide moiety (Fig. 39). It also showed no therapeutic benefits in the clinical trial against alzheimer due to poor tolerability.^244^

### 2.12. GABA

#### 2.12.1. GABA_A_ agonist

Hyperexcitation of neuronal activity is one of the toxic factors that eventually lead to neuronal death and AD progression. It was found in AD mouse models that GABAergic neurotransmission is upregulated in the hippocampus before neuronal death.^245,246^ Accordingly, it is more likely to be a significant target in order to neutralize the abnormal hyperexcitation.

Etazolate, a pyrazolopyridine derivative (Fig. 36), is a GABA_A_ receptor modulator that was found to show neuroprotective properties against the toxic effects of Aβ. Moreover, it revealed cognitive improvement and anti-inflammatory activity in traumatic brain injury.^247,248^ Further investigation into the mechanisms of its neuroprotective effect revealed GABA_A_ receptor activation as well as stimulating α-secretase cleavage of APP. The importance of its GABA_A_ role is highlighted by the full block of its neuroprotective effect by GABA_A_ antagonists.^226^

**Fig. 40:**
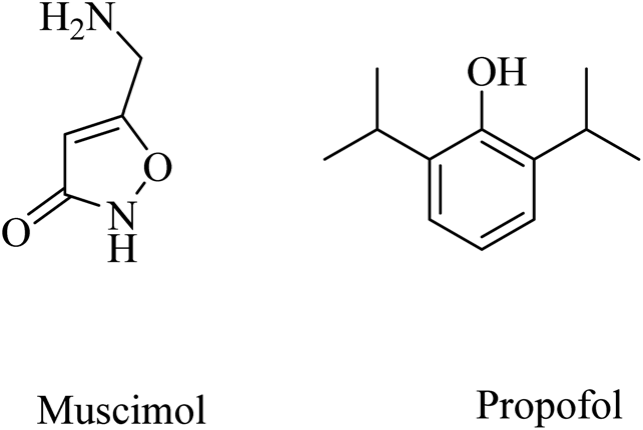
The chemical structures of some GABA_A_ agonists.

Other examples of GABA_A_ agonists that showed promising results are muscimol (5-(aminomethyl)isoxazol-3(2*H*)-one) and propofol (2,6-diisopropylphenol) (Fig. 40).^249,250^ Muscimol pretreatment was found to effectively inhibit the Aβ25–35-induced neuronal death in cultured rat cortical neurons.^250^

Long-term use of propofol for the treatment of aged mice was found to reduce Aβ40 and Aβ42 levels in the brain and decrease the expression level of BACE1, thereby decreasing the aggregation of amyloid plaques.^250^ These data reflected the importance of chronic GABA_A_ receptor activation by propofol in neuroprotection and decreasing Aβ levels in brain. Furthermore, propofol was proven to improve cognitive function in both WT and APP/PS1 mice.^249^

#### 2.12.2. GABA_B_ antagonist

In AD mouse models as well as in human AD patients, the levels of released γ-aminobutyric acid (GABA) were found to be significantly increased. The high levels of GABA could in turn bind to GABA_B_ receptors, inhibiting synaptic release in APP/PS1 mice.^245^ GABA_B_ antagonists were proposed to decrease the inhibition of synaptic function and enhance cognition in AD.^245^

**Fig. 41:**
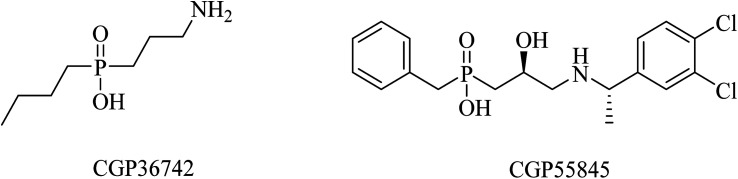
GABA_B_ antagonists.

SGS742 (CGP36742), a phosphinic acid derivative (Fig. 41), was the first GABA_B_ antagonist evaluated for AD in clinical trials. Its effects on rodents and monkeys were outstanding, as evidenced by significant improvements in cognition and learning tasks.^251^ In addition, SGS742 was found to be well tolerated not only in experimental animals but also in human volunteers. In a phase II clinical trial, 8 weeks of oral administration of SGS742 revealed considerable attention improvement and memory enhancement in patients suffering mild cognitive impairment.^251–253^

CGP55845 is another phosphinic acid-based (Fig. 41) GABA_B_ receptor antagonist. In an aged rat model with cognitive impairment, CGP55845 was found to completely improve the olfactory discrimination learning deficits and retrieve performance.^254^ These data indicated the significance of GABA_B_ receptors as a potential target for improving cognitive disorders.^255^

### 2.13. Antioxidants

#### 2.13.1. Vitamins

**Fig. 42:**
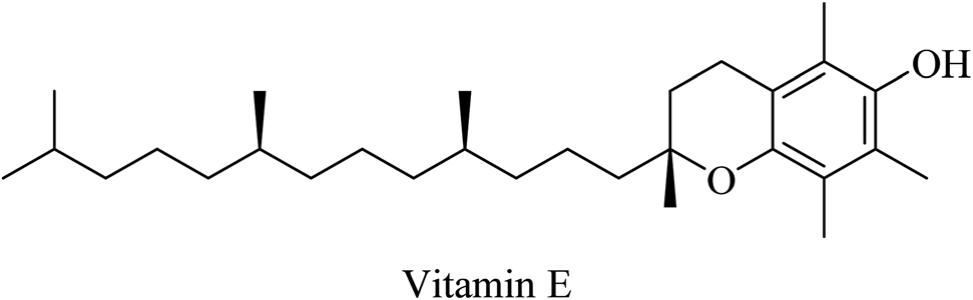
The chemical structure of vitamin E.

Vitamin E (α-tocopherol, Fig. 42) is a powerful lipid-soluble chain-breaking antioxidant, which plays a pivotal role in preventing the toxic effects of free radicals on neuronal cells and, hence, reducing the rate of progression of dementia in mammalian cells.^256^ In experimental studies, vitamin E was found to improve cognition as well as prevent the toxic effect of Aβ in rodents.^257,258^ Similar results were reported in studies conducted on AD patients.^259^ These data suggest that vitamin E, as a powerful lipid-soluble antioxidant, has the ability to significantly delay the clinical deterioration of cognitive functions in AD patients. This suggestion was supported by the finding that vitamin E considerably inhibited tau-induced neurotoxicity in Drosophila.^260^

#### 2.13.2. Natural compounds

**Fig. 43:**
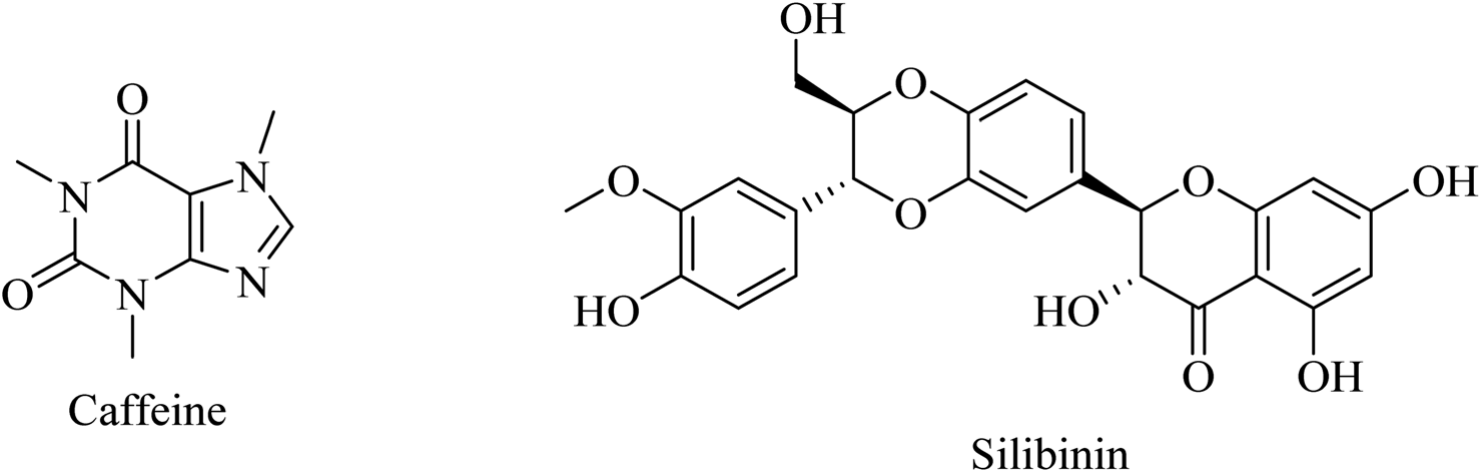
Natural antioxidants.

Caffeine (Fig. 43) is an antioxidant that was reported to inhibit amyloidosis and amyloid plaque production, reducing Aβ levels in the brain of transgenic mouse models for early-onset familial AD.^261^

Another herbal antioxidant that showed promising data with regard to improvement of AD is silibinin (silybin) (Fig. 43), a flavonoid derived from the herb milk thistle (*Silybum marianum*). Silibinin was reported to prevent memory impairment and to eliminate the oxidative stress induced by Aβ in mice, so it is likely to be a potential candidate for AD treatment.^262^

**Fig. 44:**
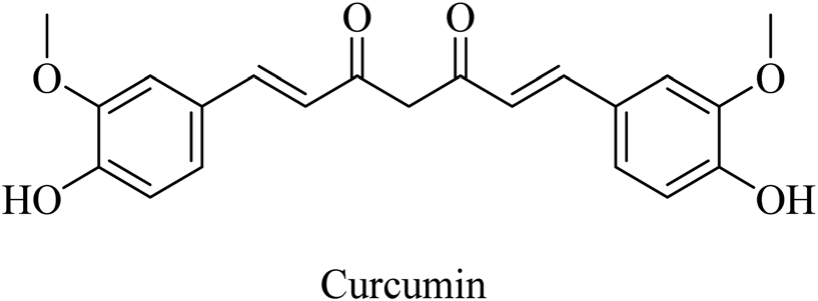
The chemical structure of curcumin.

Curcumin is a di-phenolic molecule (Fig. 44) extracted from turmeric and is known for diversity in its biological effects, such as antioxidant, anticarcinogenic, and anti-inflammatory. In the last decade, its pharmacological effects on AD have been discovered. It exhibited neuroprotection, and inhibition of Aβ aggregation, and Aβ-induced inflammation, so it is likely to be helpful in treating AD as one of the neurodegenerative diseases.^263^ Curcumin has also been found to inhibit AChE in *in vitro* studies.^263^

**Fig. 45:**
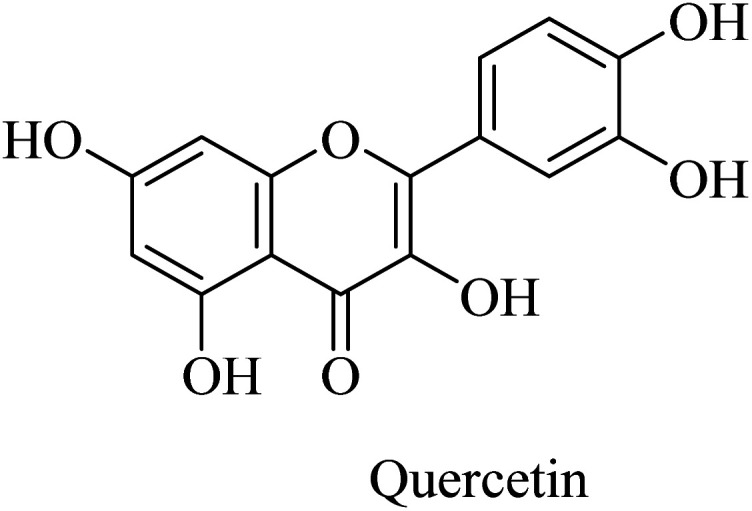
The poly phenolic quercetin.

Similarly, the polyphenolic quercetin (Fig. 45) exhibited neuroprotective properties that encourage researchers to utilize it as a lead compound for developing drugs against neurodegenerative disorders such as AD. However, the oral bioavailability of quercetin is low, so the clinical trials were impeded. It was reported to reduce β-amyloidosis, astrogliosis, tauopathy, and microgliosis in the hippocampus and amygdala.^264^

**Fig. 46:**
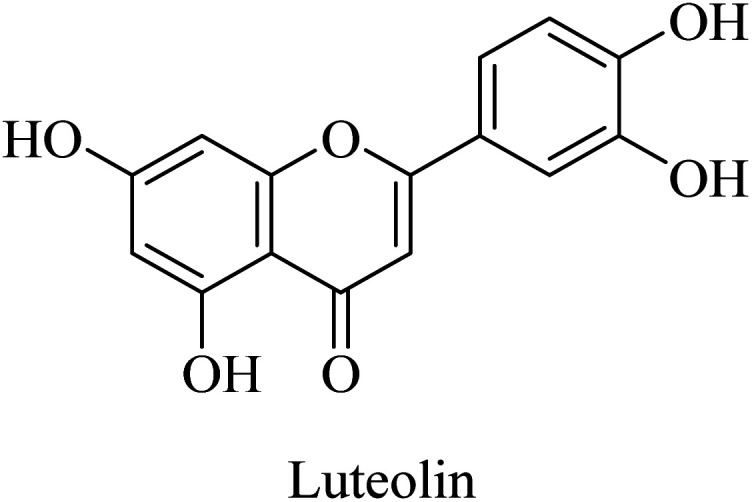
The chemical structure of luteolin.

Luteolin (Fig. 46) is a flavonoid compound that was found to act as a neuroprotective agent in a streptozotocin-induced alzheimer's rat model. This reported effect was suggested to be due to luteolin's antioxidant properties, which are mediated by blocking free radicals and dispersing amyloid plaques. Accordingly, luteolin is proposed as a potential therapeutic candidate for neuronal disorders, *e.g.*, AD; however, further investigation is still required.^265^

#### 2.13.3. Mitochondria-targeted antioxidants (MTAs)

**Fig. 47:**
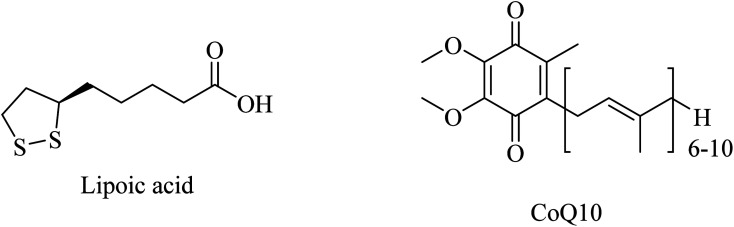
The chemical structure of lipoic acid and CoQ10.

Antioxidants such as α-lipoic acid (LA), coenzyme Q10 (CoQ10) (Fig. 47), and glutathione are likely to have a potential therapeutic effect in the treatment of some neurodegenerative diseases. Mitochondrial dysfunction was considered a proposed mechanism involved in the neuronal pathogenesis associated with some neurodegenerative diseases, such as AD. So, antioxidants that protect the mitochondria and prevent their malfunction emerged as potential therapeutic agents in many neurodegenerative diseases, including AD. Because overproduction of ROS by mitochondria is a major element in the progression of AD, many metabolic antioxidants such as LA and CoQ10 that easily penetrate the cell membrane to reach the mitochondria are more likely to provide considerable protection in AD.^261^

Administration of LA (Fig. 47) for long periods has been reported to decrease the expression of lipid peroxidation markers without reducing the Aβ levels in the brains of AD mice. Additionally, LA was found ineffective in improving cognition.^266^

CoQ10 (Fig. 47) plays a protective role, preventing mitochondrial damage by oxidative stress as well as protecting the whole neuronal cell through reducing Aβ overproduction and intracellular neurofibrillary tangles. Furthermore, CoQ10 is an essential factor for the production of ATP by mitochondria, so it is recommended as a significant antioxidant for AD prevention.^261^

#### 2.13.4. Other antioxidants

**Fig. 48:**
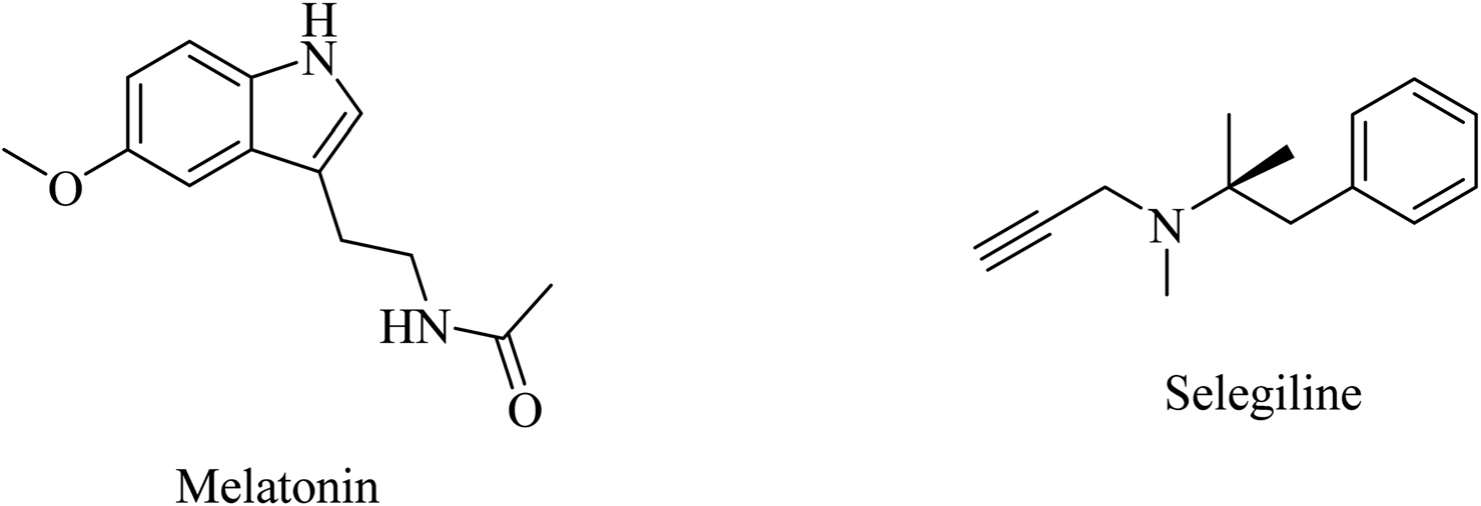
The chemical structure of melatonin and selegiline.

Melatonin (Fig. 48) investigation suggested that the antioxidant properties of melatonin could play a role in inhibiting Aβ-induced toxicity^267^ and reducing tau hyperphosphorylation.^268–271^ Furthermore, melatonin was examined in an APP695 transgenic mouse model and found to improve memory and learning deficits. Additionally, melatonin reduced Aβ-induced neuronal death in AD cell models.^272^ The above data indicate that melatonin as an antioxidant is a potential therapeutic candidate for AD; however, further clinical studies remain necessary to evaluate the efficacy and safety of melatonin for AD treatment.^261^

Selegiline (Fig. 48), a selective monoamine oxidase-B inhibitor, was suggested for neurodegenerative disease treatment due to its possible antioxidant properties in addition to rapid generation of the potent vasodilator nitric oxide in cerebral blood vessels.^273^ In 1997, it was reported to protect neurons and decrease the progression rate of AD in patients with moderately severe impairment from AD.^258^ In 2000, the analysis of 15 clinical trials revealed that there was no solid evidence to identify selegiline as a potential treatment for AD.^261^

The discussed drugs are outlined in the following table (Table 1) along with their mechanisms of action and references.

**Table 1:** The name, mechanism of action, and reference of the drugs discussed above

Serial	Drug	Mechanism of action	Reference
1	Galantamine	AChEI	62
2	Donepezil	AChEI	62
3	Rivastigmine	AChEI and BChEI	63
4	Tacrine	AChEI and BChEI	65
5	Metrifonate	Irreversible AChEI and BChEI	64
6	ABT-126	Selective α7 nicotinic receptor agonist	72
7	ABT-089	Selective α4β2 nicotinic partial agonist	75
8	Oxotremorine	CNS muscarinic agonist	77
9	Xanomeline	CNS muscarinic agonist	79
10	EUK1001	CNS muscarinic agonist	79
11	Memantine	NMDA antagonist	82
12	Phencyclidine	NMDA antagonist	86
13	Vinpocetine	PDE1 inhibitor	89
14	Cilostazol	PDE3 inhibitor	87
15	HT-0712	PDE4 inhibitor	96
16	Roflumilast	PDE4 inhibitor	96
17	Zatolmilast (BPN14770)	PDE4 inhibitor	96
18	Denbufylline	PDE4 inhibitor	99
19	Sildenafil	PDE5 inhibitor	87 and 96
20	BI-409306	PDE9 inhibitor	100 and 101
21	PF-04447943	PDE9 inhibitor	100 and 101
22	Propentofylline	Broad-spectrum PDE inhibitor	87
23	Aducanumab	Selective anti-Aβ monoclonal antibody	108–112
24	Lecanemab	Selective anti-Aβ monoclonal antibody	113–116
25	Solanezumab	Monoclonal anti-Aβ antibody	118 and 119
26	Bapineuzumab	Monoclonal anti-Aβ antibody	120 and 121
27	Ponezumab	Monoclonal anti-Aβ antibody	122
28	Gantenerumab	Monoclonal anti-Aβ antibody	119
29	AN1792	Purified Aβ-42 polypeptide vaccine	125
30	ACC-001	N-terminal Aβ1-7 vaccine	57
31	QS-21	Adjuvant combined with ACC-001	129 and 130
32	ABvac40	C-terminal end of Aβ40 vaccine	57
33	Tramiprosate	Inhibitor for Aβ42 oligomer formation	133–136
34	Valitramiprosate (ALZ-801)	Inhibitor for Aβ42 oligomer formation (prodrug)	133
35	Sodium selenate	PP2A activator	140
36	Methylthioninium chloride	PP2A activator	142
37	Tideglusib	GSK3β inhibitor	145 and 146
38	Lithium chloride	GSK3β inhibitor	150
39	LY2811376	BACE-1 inhibitor	151
40	LY2886721	BACE-1 inhibitor	16 and 153
41	Umibecestat (CNP520)	BACE-1 inhibitor	155
42	Verubecestat (MK8931)	BACE-1 inhibitor	158 and 159
43	Atabecestat (JNJ-54861911)	BACE-1 inhibitor	161–163
44	Lanabecestat	BACE-1 inhibitor	167
45	Elenbecestat (E2609)	BACE-1 inhibitor	169
46	NB-360	Dual BACE-1 and BACE-2 inhibitor	171
47	RG712	Dual BACE-1 and BACE-2 inhibitor	174
48	Semagacestat (LY450139)	γ-Secretase inhibitor	178
49	MK-0752	γ-Secretase inhibitor	154
50	Avagacestat (BMS-708163)	Selective γ-secretase inhibitor	182
51	Begacestat (GSI-953)	Selective γ-secretase inhibitor	186
52	PF-3084014	Notch sparing γ-secretase inhibitor	189 and 190
53	Ibuprofen	First generation GSM	194 and 195
54	Indomethacin	First generation GSM	194 and 195
55	Sulindac	First generation GSM	194 and 195
56	Tarenflurbil	Second generation GSM	197
57	JNJ-40418677	Second generation GSM	202
58	EVP-0015962	Second generation GSM	203
59	Itanapraced (CHF5074)	Second generation GSM	205
60	BIIB042	Second generation GSM	203 and 208
61	E2012	Second generation GSM	154 and 210
62	E2212	Second generation GSM	211
63	BMS-869780	Second generation GSM	214
64	BMS-932481	Second generation GSM	154
65	PF-06648671	Second generation GSM	215
66	SPI-014	Second generation GSM	217
67	SPI-1865	Second generation GSM	217
68	Dihydroergocristine	Second generation GSM	219
69	Acitretin	α-Secretase activator	223
70	Etazolate (EHT-0202)	GABA_A_ modulator & α-secretase activator	225
71	PF-05212377 (SAM-760)	Selective 5-HT6R antagonist	57 and 231
72	Iadalopirdine	Selective 5-HT6R antagonist	233
73	Varoglutamstat (PQ-912)	QC inhibitor	235
74	Etanercept	Competitive TNF-α inhibitor	243
75	Thalidomide	Immunomodulator & TNF-α inhibitor	244
76	Muscimol	GABA_A_ agonist	250
77	Propofol	GABA_A_ agonist	250
78	SGS742 (CGP36742)	GABA_B_ antagonist	251
79	CGP55845	GABA_B_ antagonist	254
80	Vitamin E	Antioxidant	257 and 258
81	Caffeine	Antioxidant	261
82	Silibinin	Antioxidant	262
83	Curcumin	Antioxidant	263
84	Quercetin	Antioxidant	264
85	Luteolin	Antioxidant	265
86	α-Lipoic acid	Mitochondrial-targeted antioxidant	261 and 266
87	Coenzyme Q10	Mitochondrial-targeted antioxidant	261
88	Melatonin	Antioxidant	267–271
89	Selegiline	Antioxidant	258 and 261

## 3. Conclusion and perspectives

Herein, AD potential therapeutic targets have been discussed, along with the clinically studied relevant drugs. As can be seen above, only a few drugs have been approved for the treatment of AD. Galantamine, donepezil, and rivastigmine (ChEIs), memantine (NMDA antagonist), and aducanumab and lecanemab (selective anti-Aβ monoclonal antibodies) are the currently approved drugs. This limited number of clinically used drugs against AD does not reflect the large number of proteins and enzymes identified as significant therapeutic targets of AD or the extensive number of drugs studied in the clinical trials of AD. But this image reflects the complexity of the disease and the lack of concrete evidence for the exact, definite cause of the disease.

Accordingly, AD can be considered a multifactorial disease that requires a deeper understanding of its etiology to give priority to the most crucial targets. At the same time, early diagnosis of the disease is of great importance to maximize the benefits of the treatment; especially, it was found that amyloid plaques and neurofibrillary tangles can be detected decades before the appearance of symptoms.^274^ As we saw earlier, lecanemab has been approved for the early stages of AD. So, the identification of early detectable biomarkers of AD is highly significant. Furthermore, there is some evidence that the approach to prevent further formation of amyloid plaques is not enough for the treatment of AD in the sense that the neurodegeneration is triggered by the neurotoxic effects of the already aggregated Aβ. This may provide an explanation for the failure of almost all clinical trials based on the prevention of Aβ aggregation. So, all aspects should be taken into account in any further clinical study. The clinical data given in this work reveals GSM and QC inhibitors as the most promising classes involving inhibition of the formation of toxic Aβ. Additionally, α-secretase activators enhance proteolysis of APP in the non-amyloidogenic pathway. At the same time, the current study presented many therapeutic classes that have been proven to protect neurons from the toxic effects of Aβ. They include the neuroprotective GABA_A_ agonists, antioxidants, anti-inflammatory, and immunomodulatory. Furthermore, the significant effects of PP2A activators and GSK-3β inhibitors in preventing neurofibrillary tangles should also be considered. For future work, it may be beneficial in such a case to apply molecular hybridization in order to develop potential drugs that work on more than one target linked firmly to AD. Otherwise, a combination of AD drugs is highly recommended. Clinical trials of drugs acting on Aβ and tau protein in combination with neuroprotective agents may change the current situation and reveal a significant protocol for AD treatment.

For the design of new anti-alzheimer small molecules, two isosteric nuclei showed very significant therapeutic properties, including reduction of the production of further toxic Aβ, activation of the cleavage of APP to soluble Aβ rather than the insoluble one, neuroprotection against the toxic effects of accumulated Aβ, and symptomatic improvement in cognitive functions. These two promising isosteric nuclei are benzimidazole and pyrazolopyridine. We saw above that their derivatives revealed QC inhibition, α-secretase activation, GABA_A_ modulation, and 5HT antagonist activity, showing very promising clinical results. The importance of these nuclei was highlighted by the therapeutic effects of their isostere, xanthine nucleus. We found that xanthine derivatives exhibited PDE inhibition as well as antioxidant activity. The most significant of them is benzimidazole in the sense that imidazole is considered a zinc binding group in QC inhibition, and on it a class of anti-alzheimer drugs were built. This class is called non-NSAID-derived imidazole GSMs, as explained earlier. Accordingly, these nuclei can be used as a scaffold for building new candidates for potential multi-target anti-alzheimer activity, taking together the clinical results and molecular structure of their derivatives discussed in the current study.

## Conflicts of interest

There is no any conflict of interest.

## Supplementary Material
